# Biostimulation of Indigenous Microbial Community for Bioremediation of Petroleum Refinery Sludge

**DOI:** 10.3389/fmicb.2016.01407

**Published:** 2016-09-21

**Authors:** Jayeeta Sarkar, Sufia K. Kazy, Abhishek Gupta, Avishek Dutta, Balaram Mohapatra, Ajoy Roy, Paramita Bera, Adinpunya Mitra, Pinaki Sar

**Affiliations:** ^1^Department of Biotechnology, Indian Institute of Technology Kharagpur, India; ^2^Department of Biotechnology, National Institute of Technology Durgapur, India; ^3^School of Bioscience, Indian Institute of Technology Kharagpur, India; ^4^Department of Agricultural and Food Engineering, Indian Institute of Technology Kharagpur, India

**Keywords:** petroleum refinery sludge, microbial community, bioremediation, biostimulation, next generation sequencing

## Abstract

Nutrient deficiency severely impairs the catabolic activity of indigenous microorganisms in hydrocarbon rich environments (HREs) and limits the rate of intrinsic bioremediation. The present study aimed to characterize the microbial community in refinery waste and evaluate the scope for biostimulation based *in situ* bioremediation. Samples recovered from the wastewater lagoon of Guwahati refinery revealed a hydrocarbon enriched [high total petroleum hydrocarbon (TPH)], oxygen-, moisture-limited, reducing environment. Intrinsic biodegradation ability of the indigenous microorganisms was enhanced significantly (>80% reduction in TPH by 90 days) with nitrate amendment. Preferred utilization of both higher- (>C30) and middle- chain (C20-30) length hydrocarbons were evident from GC-MS analysis. Denaturing gradient gel electrophoresis and community level physiological profiling analyses indicated distinct shift in community’s composition and metabolic abilities following nitrogen (N) amendment. High throughput deep sequencing of 16S rRNA gene showed that the native community was mainly composed of hydrocarbon degrading, syntrophic, methanogenic, nitrate/iron/sulfur reducing facultative anaerobic bacteria and archaebacteria, affiliated to γ- and δ-*Proteobacteria* and *Euryarchaeota* respectively. Genes for aerobic and anaerobic alkane metabolism (*alk*B and *bss*A), methanogenesis (*mcr*A), denitrification (*nir*S and *nar*G) and N_2_ fixation (*nif*H) were detected. Concomitant to hydrocarbon degradation, lowering of dissolve O_2_ and increase in oxidation-reduction potential (ORP) marked with an enrichment of N_2_ fixing, nitrate reducing aerobic/facultative anaerobic members [e.g*., Azovibrio*, *Pseudoxanthomonas* and *Comamonadaceae* members] was evident in N amended microcosm. This study highlighted that indigenous community of refinery sludge was intrinsically diverse, yet appreciable rate of *in situ* bioremediation could be achieved by supplying adequate N sources.

## Introduction

*In situ* bioremediation of highly hazardous petroleum refinery wastes containing different types of aliphatics, aromatics, other complex hydrocarbons and heavy metals is a technological challenge worldwide (Das and Chandran, 2011; Ivshina et al., 2015; Pal et al., 2016). Considerable work on microbiology of diverse hydrocarbon rich environments (HREs) have demonstrated natural abundance of hydrocarbon degrading microorganisms, particularly, bacteria and archaebacteria capable of degrading wide range of hydrocarbons as nutritional resources (Alonso-Gutiérrez et al., 2009; An et al., 2013; Hazen et al., 2013; Head et al., 2014; Fowler et al., 2016). Yet, the success of *in situ* bioremediation using the indigenous microorganisms remain severely constrained by inappropriate nutrient level and/or physico-chemical conditions (viz., temperature, pH, moisture content, nutrient availability, etc.) prevailing at the contaminated sites (Lu et al., 2014; Smith et al., 2015). Particularly, paucity of essential nutrients [e.g., nitrogen (N), phosphorous (P), terminal electron acceptor (TEA), etc.] have often been found to be the critical factor in reducing the rate of microbial metabolism and thus, natural attenuation. The lack of appropriate and readily available nutrient for proper utilization of hydrocarbon substrates necessitates engineered bioremediation strategies including biostimulation. Successful bioremediation events have been reported for various hydrocarbon contaminated sites, wherein, amendment of appropriate inorganic nutrients (N and/or P) resulted in enhanced growth and activity of efficient indigenous microorganisms, thus, expediting bioremediation (Stroud et al., 2007; Smith et al., 2015; Zhang and Lo, 2015). N (in the form of nitrate) amendment has been found to be one of the most efficient biostimulation approach, owing to thermodynamic favorability of nitrate as TEA which facilitates efficient oxidation of carbon substrates, allowing bacterial growth as well as hydrocarbon catabolism (Dashti et al., 2015; Bell et al., 2016).

Characterization of the indigenous microbial communities, in terms of their diversity, metabolic potential and response (change in community composition) toward biostimulatory agents, is essential for developing bioremediation technology. Culture-independent molecular techniques have identified diverse and complex assemblages of aerobic and anaerobic bacteria and archaea capable of hydrocarbon degradation, nitrate/sulfate/iron-reduction, fermentation, as well as methane metabolism in various HREs (An et al., 2013; Daffonchio et al., 2013; Hazen et al., 2013; Hu et al., 2013; Das and Kazy, 2014; Head et al., 2014). Catabolic potential of microbial communities in HREs, as elucidated by gene specific or shotgun metagenome or microarray based analyses, revealed co-occurrence of genes related to aerobic and anaerobic biodegradation of hydrocarbons and associated nutrient metabolism. Such association of aerobic and anaerobic organisms might be contributing in the concerted metabolic interplay for biodegradation of hydrocarbons in a complex environment.

To gain a better insight into the nature of indigenous microbial community within petroleum refinery waste and evaluate the prospect for *in situ* bioremediation by biostimulation, we have adopted a microcosm based culture-independent metagenomic approach. In the present work, we have investigated the effect of biostimulation on biodegradation potential as well as change in microbial community composition of petroleum hydrocarbon containing sludge of Guwahati Oil refinery, Assam, India. To elucidate the role of nitrate addition in enhancing biodegradation and identifying the microbes responsible for the process, prokaryotic diversity within the native and biostimulated communities was analyzed using high throughput deep sequencing of 16S rRNA genes. Analysis of genes encoding key enzymes in the metabolic cycling in petroleum contaminated sludge was performed. The overall study was organized to answer the following key questions: (i) what is the composition of autochthonous microbial community within oil refinery waste sludge, (ii) how far different biostimulation approaches can improve intrinsic hydrocarbon biodegradation potential of the community and (iii) what is the effect of specific biostimulation strategy on the indigenous microbial community composition and metabolic activities? The study elucidates a detailed composition of microbial community residing in refinery waste sludge and explores the scope for *in situ* bioremediation.

## Materials and Methods

### Sample Collection, Physicochemical Characterization and Enumeration of Microbial Cells

Petroleum refinery waste sludge was collected from sludge waste lagoon of the oil refinery owned by Indian Oil Corporation Limited (IOCL) at Guwahati, Assam, India (26.14° N, 91.73° E) using sterile spoons and stored at 4°C. Physicochemical parameters including pH, temperature, dissolved oxygen (DO), oxidation reduction potential (ORP) and conductivity of the samples were measured on-site using an Orion Star 140^TM^ series meter (Thermo Electron Corporation, Beverly, MA, USA) (analysis for three aliquots done in triplicate for each parameter). Detailed chemical characterization of the sample was done with respect to its total organic carbon (TOC), total N (as nitrate and 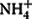), total phosphate, moisture, total petroleum hydrocarbon (TPH), and oil and grease content using standard procedures (Walkley and Black, 1934; Cataldo et al., 1975; Baethgen and Alley, 1989). Heavy metal concentrations were determined by inductively coupled plasma-mass spectrophotometer (ICP-MS) (Varian 810 ICP-MS System, USA). Major anions were estimated using ion chromatograph (Dionex, USA). Characterization of hydrocarbon constituents was ascertained by Gas Chromatograph coupled with Mass Spectrometer (GC-MS) (Perkin Elmer GC-MS) following extraction from the oily sludge sample with *n-*hexane (Sigma–Aldrich, HPLC grade) in a 1:10 (sample: solvent) ratio. Aerobic and anaerobic culturable bacterial populations were enumerated on Reasoner’s 2A (R2A) agar medium and anaerobic agar (HiMedia, India). For bacterial counts sludge sample was suspended in 0.85% sterile saline, vortexed to mix properly, 100 μl of serially diluted suspensions were plated on agar medium and incubated at 30°C for 7 (aerobic) or 30 (anaerobic) days. Anaerobic growth was checked under nitrogen purged anaerobic environment in an anaerobic chamber (Coy Laboratory Products Inc.) and incubated in anaerobic jar (HiMedia).

### Microcosm Set Up

Laboratory microcosms were set up in cotton plugged serum vials (27 ml; Sigma–Aldrich, USA) with 5 g sludge and various amendments. N [as nitrate (NaNO_3_), 10 mM], P [as phosphate (K_2_HPO_4_) 0.16 mM] or S [surfactant (Tween 80, 1% w/v)] was used either individually (as N-, P-, S- amendment) or in combination (NP, NS, PS, NPS) (**Table 1**). Sterile water was added to each set of microcosms to achieve a moisture content of 60% (Dibble and Bartha, 1979). The concentrations of N and P were chosen to maintain a final C:N:P ratio of 100:10:1 within the sample. To estimate abiotic losses (of hydrocarbons) as well as intrinsic biodegradation potential of the sludge community, microcosm without any amendment; killed (K) and unamended (U) controls were used. To kill the indigenous microbial community 1% (w/v) sodium azide was used as a biocide (Adetutu et al., 2012). All microcosms were prepared in triplicates for each time point and each treatment. Total 9 (treatments) × 7 (time points) × 3 (triplicates) = 189 vials were set up and incubated aerobically at room temperature (27°C) with occasional shaking. Samples were collected from sacrificial serum vials (triplicate) at 15 days interval for 3 months and analyzed. Additional microcosms were prepared subsequently with (a) varying N concentration and (b) considering different sludge samples collected from other refinery or oil exploration site. Details of these microcosm preparations were same as described before, except otherwise mentioned (in Result section).

**Table 1:** Microcosm setup for biodegradation of petroleum hydrocarbons.

Microcosm designation	NaNO_3_ as N source	K_2_HPO_4_ as P source	Surfactant (Tween 20)	Treatment
K	-	-	-	Control to account for abiotic losses
U	-	-	-	Control for natural attenuation
N	+	-	-	N amendment
P	-	+	-	P amendment
NP	+	+	-	N and P amendment
Surf	-	-	+	Surfactant as an aid to bioremediation
NS	+	-	+	N and surfactant amendment
PS	-	+	+	P and surfactant amendment
NPS	+	+	+	Biostimulation (N and P) and surfactant


Samples withdrawn from the microcosms at periodic intervals were used to monitor several parameters including number of the viable cells [as colony forming units (CFUs) on R2A agar plate], CLPP and TPH content. DGGE as well as next generation sequencing of the 16S rRNA gene of selected samples was performed with extracted metagenomes to assess possible change in microbial community composition during the biostimulation. Selected physiochemical parameters (viz., ORP, DO, pH, and conductivity), were measured following 90 days incubation using ion selective probes and Orion Star 140^TM^ series meter.

### Enumeration of Cell Viability, Dynamics of Community Composition and Physiological Characterization of the Communities within Microcosms

Viability of indigenous bacterial cells within the sludge samples withdrawn from the microcosms was monitored by quantifying the CFUs g^-1^. One gram of sludge sample was mixed with 9 ml of sterile 0.85% (w/v) NaCl and 1% (w/v) sodium pyrophosphate, vortexed for 1 min, incubated in an incubator shaker for 30 min at 170 rpm at 30°C. This suspension was further diluted serially to the required concentration and plated on R2A agar plate in duplicate and incubated for 48–72 h at 30°C for heterotrophic bacterial plate count.

Dynamics of microbial community was studied using DGGE. Metagenomic DNA was extracted from all the microcosm samples (eight aliquots from each sample) using MoBio PowerSoil DNA extraction kit (MoBio Laboratories, Carlsbad, CA, USA), quantified through Nano Drop spectrophotometer (Thermo Scientific, USA) and V3 regions of 16S rRNA gene were amplified using GC-clamped 341F and 518R primers. A DCode Universal Mutation Detection System (Bio-Rad, USA) was used to separate the PCR amplified 16S rRNA gene fragments by DGGE as described in Paul et al. (2015).

Physiological potential of microbial communities from different microcosms was assessed by community level physiological profiling system (Biolog ECO plates). Utilization of 31 different substrates by the sludge community was estimated over 7 days at 12 h interval using Biolog ECO plates. Dislodging of cells from the sludge was done using 1% (w/v) sodium pyrophosphate (Wang et al., 2012) and resulting cell suspension (herein referred as Sludge Liquid Extract or SLE) was used. Viable cell number in the extract was determined by CFU count on R2A medium. 150 μL of this SLE was inoculated in each well of a Biolog ECO plate (incubated at 30°C) and optical density of the wells (at 590 nm) was observed at an interval of every 12 h. The average well color development (AWCD), Shannon–Weiver index (H) and Simpson’s diversity index (D) were estimated according to the following equations:


$$AWCD=\overset{31}{\underset{i=0}{\Sigma }}{OD}_{i}/31$$

$$Shannon-Weiner\,\;diversity\,\;index\,\;\left(H^{\prime }\right)=-\overset{31}{\underset{i=0}{\Sigma }}p_{i}\,\;In\,\;{\;p}_{i}$$

$$Shannon\prime s\,\;diversity\,\;index\,\;\left(D\right)=1-\overset{31}{\underset{i=0}{\Sigma }}{p_{i}}^{2}\,$$

### Estimation of Hydrocarbon Degradation in Microcosms

Biodegradation of hydrocarbons was monitored by gas chromatography equipped with flame ionization detector (GC-FID, Clarus 580, Perkin Elmer, USA) using the *n*-hexane extractable fraction of the sludge. Samples were extracted following EPA method 3530. Briefly, 1 g sludge was extracted twice with 10 ml *n*-hexane, vortexed thoroughly for 30 min and centrifuged at 10,000 rpm for 10 min to separate the particulates. TPH content was estimated by injecting this extract in GC using the method as described by Wallisch et al. (2014). Quantification of the amount of TPH present in each sample was achieved by summing up unresolved and resolved components of the hexane extract between retention times 3–30 min (for C_10_H_22_ to C_40_H_82_). Relative abundance of different carbon chain length compounds in the samples was further estimated by comparing retention times of peaks corresponding to standard n-alkanes (obtained from Sigma Aldrich, USA, TPH mix 3) (Zhang et al., 2015). Percentage TPH reduction was estimated by the following equation:


$$Percentage\,\;reduction=\frac{initial\,\;TPH-final\,\;TPH}{initial\,\;TPH}\times 100$$

Extracts from selected samples showing considerable TPH degradation (>70%) were filtered through glass wool and analyzed in a Shimadzu QP2010SE GC-MS system to identify the composition of the constituent hydrocarbons. For separation of the components, a ZB-5 column (30 m × 0.25 mm id, film thickness 0.25 μm) was used along with helium as a carrier gas. Two microliter of the extract was injected with split ratio 2: 2. The injector temperature was set at 260°C and the column flow rate was maintained at 1 ml min^-1^. The column oven temperature program was set at 50°C for 2 min, then increased to 60°C at a rate of 2°C min^-1^ hold for 2 min, subsequently raised to 210°C at 3°C min^-1^ and hold for 2 min, finally to 270°C at 10°C min^-1^ and hold for 7 min. The conditions for the operation of mass spectrometer were set as follows: ion source temperature, 200°C; interface temperature, 280°C; electron energy, 70 eV; scanning range of m/z, 40–600 a.m.u. Identification of the components was carried out by comparing mass spectrum of the component to that of mass spectral library from NIST 05 (National Institute of Standards and Technology, Gaithersburg, USA) and Wiley 8.0.

### Analysis of Microbial Community Structure and Composition in Microcosms Using Metagenome Sequencing

Microbial diversity and community composition within the sludge samples [native sludge sample, GR3 and three microcosm derived samples (N, NS and U)] were studied by next generation sequencing (NGS) based approach. Total metagenome obtained (using the method as described in earlier section) in various replicates was pooled and V4 region of 16S rRNA gene was amplified using universal V4 specific fusion bacterial primers (barcoded for multiplexing) to generate amplicon library. Single-end sequencing of the V4 region was performed in a 318 Ion Express chip in a Personal Genome Machine, Ion Torrent (Life Technologies). Raw reads obtained from the Ion torrent platform was analyzed through QIIME version 1.7.0 (Quantitative Insights In to Microbial Ecology) pipeline (Caporaso et al., 2010). Raw reads were merged together for GR3 (as it was sequenced twice) for further processing in QIIME. Quality filtering was performed by removing barcode, primer sequences, sequences with homopolymers run of >6 bp and reads having sequence length less than 250 bp. Operational taxonomic units (OTUs) were identified at the sequence identity level of 97% using pick_otus.py command with UCLUST algorithm. Afterward, a representative sequence was selected from each OTU using default parameters in QIIME and the taxonomic assignment was attained though the command assign_taxomony.py using RDP classifier trained with Greengenes database 13.8 with a minimum confidence of 80%. PyNAST tool was used to align the sequences using the command align_seqs.py with default parameters and filter_alignment.py command was used to filter sequence alignment by removing highly variable region. Chimeric sequences were identified through Chimera slayer using identify_chimeric_seqs.py command. Finally, the script make_otu_table.py was used to prepare the OTU table (excluding chimeric sequences). The alpha diversity of all the sample, viz., Chao1, Shannon and Simpson indices, Good’s coverage and Observed species, was estimated using alpha_diversity.py command with set default parameters. Rarefaction curve was drawn using alpha_rarefaction.py and UPGMA based on Weighted Unifrac among four samples was drawn using jackknifed_beta_diversity.py. The raw reads were deposited to the NCBI Sequence Read Archieve database (SRA) under accession number SAMN04122422, SAMN04122421, SAMN04122412, SAMN04122411, SAMN04122402 (Bioproject ID:PRJNA297334).

### Amplification of Functional Genes from Selected Microcosm Samples

Presence of major functional genes related to denitrification, N_2_ fixation, sulfate reduction and hydrocarbon degradation within the community was ascertained by PCR based approach. Metagenomes from the GR3 and N amendment samples were used as template for amplification of target functional genes such *as nar*G, *nrf*A, *nir*S, *nif*H, *mcr*A, *alk*B, *dsr*B and *bss*A like gene (fumarate adding motif of this gene). Details of the primers used for each gene and PCR conditions are presented in **Table 2**. Amplified products were eluted from 1.5% agarose gel, cloned in pTZ57R/T vector (InsTAclone^TM^ PCE cloning kit, Thermo Scientific) and sequenced (Eurofins, Bangaluru, India) using primer specific M13 forward primer. The sequence reads were translated using ExPASy tools^1^ and homology to related gene product sequences was ascertained using BLASTP of NCBI database. Identification of the clone sequences were determined by BLASTN and sequences with the closest identity were retrieved from NCBI. These sequences were aligned using CLUSTALW and phylogenetic trees were constructed using Neighbor joining algorithm with 1000 bootstrap repetitions in MEGA 6 (Tamura et al., 2013).

**Table 2:** Details of the primers used with PCR cycle conditions^∗^.

Gene	Primers (5′-3′)	Size (bp)	Annealing temperature (°C)	Source
alkB	F(AAYACNGCNCAYGARCTNGGNCAYAA)	550	53	Kloos et al., 2006
	R(GCRTGRTGRTCIGARTGICGYTG)			
bssA	7772f (GAC ATG ACC GAC GCS ATY CT)	794	50–55	Winderl et al., 2007
	8546r (TCG TCG TCR TTG CCC CAY TT)			
*mcr*A	ME1(GCMATGCARATHGGWATGTC)	790	50–55	Hales et al., 1996
	ME2(TCATKGCRTAGTTDGGRTAGT)			
*nif*H	H3(ATR TTR TTN GCN GCR TA)	330	45–50	Zehr and McReynolds, 1989
	H4(TTY TAY GGN AAR GGN GG)			
*nir*S	S1F(CCTAYTGGCCGCCRCART)	890	40–45	Braker et al., 1998
	S6R (CGTTGAACTTRCCGGT)			
*dsr*B	p2060F (CAACATCGTYCAYACCCAGGG)	450	55	Wagner et al., 1998; Geets et al., 2006
	4R (5′-GTGTAGCAGTTACC GCA-3′)			
*nar*G	1960f (TAYGTSGGCCARGARAA)	650	55–59	Philippot et al., 2002
	2659r (TTYTCRTACCABGTBGC)			
*nrf*A	F1(GCNTGYTGGWSNTGYAA)	520	50–55	Smith et al., 2007
	7R1 (TWNGGCATRTGRCARTC)			


*^∗^All PCR reactions were carried out with denaturation at 94°C for 5 min, followed by 35 cycles of initial denaturation at 94°C for 45 s, 30 s at respective annealing temperature and extension at 72°C for 1 min (in case of gene size > 700), 45 s (for gene size < 700) and a final extension at 72°C for 10 min.*

### Statistical Analysis

The data were subjected to one-way analysis of variance (ANNOVA) at 5% probability. Mean of the different treatments were tested for level of significant differences at *p* < 0.05 by Tukey (Honestly Significant Difference) test. Associations between variables were calculated by Pearson’s correlation. Bray–Curtis similarity on relative abundance of families among the treated and untreated samples was used to plot UPGMA (Unweighted Pair Group Method with Arithmetic Mean Analysis) using MVSP 3.2 software. Significant difference of the microbial assemblages and other parameters derived from both treated and control were analyzed for principal coordinates analysis (PCA) using MVSP 3.2 software. Differences were considered significant at *p* < 0.05.

## Results

### Physicochemical and Microbial Characterization of the Sample

Physicochemical properties of the GR3 sample were presented in **Table 3**. Presence of low moisture and dissolve oxygen (DO), negative ORP, high conductivity and slightly acidic (pH 6.0–6.5) condition was noted. Temperature of the sample was found to be higher (45°C) than the average temperature of this region (39°C). Among the anions tested, concentration of chloride (469 mg kg^-1^) was significantly higher (*p* < 0.05) than the others detected. Sulfate and phosphate were present in relatively higher concentrations over that of nitrate and nitrite. Heavy metals like iron and zinc were detected at considerably higher concentrations (130–300 mg kg^-1^), while arsenic, cadmium, cobalt, chromium, lead and nickel were present at relatively lower concentrations. TPH content was high and mostly (75%) constituted by aliphatic and some (12%) aromatic fractions. The analysis of length distribution of carbon chains revealed an abundance of C8–C16 compounds. GC-MS analysis further ascertained the presence of moderate chain length aliphatic compounds (e.g., pentadecane, dodecane, hexadecane, non-adecane, etc.,) and cyclohexane. Presence of naphthalene and its derivatives, as well as methylphenol compounds, were also evident, but to lesser extent.

**Table 3:** Physico-chemical and microbiological characterization of oily sludge sample.

Parameters	Values
pH	6–6.5
Temperature (°C)	44.9
Dissolved oxygen (mg/l)	0.19
Oxidation reduction potential (mV)	-30
Conductivity (μ Siemens/cm)	1.4
Moisture content (%)	9
Oil and gas content (%, w/w)	90.3
Total organic carbon (TOC) (g/kg)	200 ± 105.2
TPH (g/kg)	400 ± 47.22
Nitrate (mg/kg)	7.875 ± 4.62
Ammonium (mg/kg)	17 ± 5.33
Nitrite (mg/kg)	<2.0
Chloride (mg/kg)	469.85
Sulfate (mg/kg)	73.61
Phosphate (mg/kg)	35 ± 10

**Heavy metals**	

As (mg/kg)	1.279 ± 1.25
Cd (mg/kg)	0.16 ± 1.91
Co (mg/kg)	2.39 ± 1.68
Cr (mg/kg)	8.07 ± 1.39
Fe (mg/kg)	302.97 ± 2.56
Na (mg/kg)	2.23 ± 2.11
Ni (mg/kg)	7.11 ± 1.63
Pb (mg/kg)	4.02 ± 2.5
V (mg/kg)	2.84 ± 1.69
Zn (mg/kg)	131 ± 2.02

**Microbial counts (CFU gram^-1^)**	

Aerobic in R2A medium	5–8 × 10^6^
Anaerobic agar medium	2 × 10^2^

**Hydrocarbon composition**	

Aliphatics (%)	95.52
Aromatics (%)	4.48


Cultivable aerobic and anaerobic bacterial counts as determined by CFU estimation on different media revealed nearly 5 × 10^6^ cells gm^-1^ sample on R2A agar medium and 2 × 10^2^ cells gm^-1^ sample (values based on wet sludge) on anaerobic agar medium indicating considerable abundance of aerobic and anaerobic microorganisms in the sample.

### Biodegradation Experiment Using Microcosms

#### TPH Degradation

**FIGURE 1:**
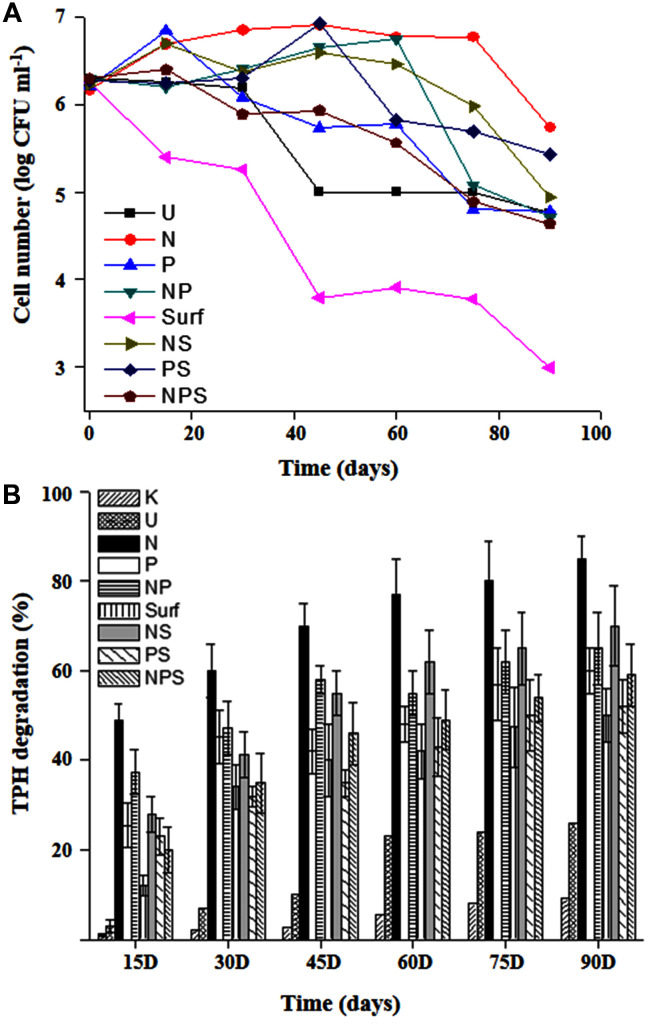
**Hydrocarbon degradation profile and cell viability in microcosms.** Cell viability **(A)** and TPH biodegradation **(B)** profiles of the sludge community undergoing different treatments during microcosm based biostimulation experiment. For legend details refer **Table 1**.

A microcosm based approach was adopted to assess the intrinsic biodegradation potential of the community and scope for its biostimulation with nutrient amendment. During the course of incubation within the microcosm set up, cell viability was monitored at different time intervals (**Figure 1A**). Except the killed control, CFU counts revealed presence of viable cells in all sets, although their numbers varied significantly among the treatments. CFU counts indicated an increase in cell number till 30 days in all the treatments except those devoid of any amendment or with surfactant amendment (individually or in combination with both N and P). Surfactant amendment led to a gradual decrease in cell viability over time throughout the incubation period. Unamended control set (U) maintained the viable cell counts till 30 days which gradually showed loss in subsequent phase over time. It was observed that number of viable cells either remained consistent or increased with various amendments over varying time spans which may be summarized as N (75 days), NP and NS (60 days), PS (45 days), P and NPS (15 days).

The extent and kinetics of TPH degradation were monitored within all the microcosms till 90 days (**Figure 1B**). U-microcosm (U control set) showed the baseline attenuation (22%) of constituent hydrocarbons till 60 days, while an extended incubation resulted in marginal increase. Among the treatments, N amendment facilitate significant (*p* < 0.05) enhancement of TPH degradation within 15 days and the trend continued till the end of the experiment yielding up to 85% degradation in 90 days. P and NP amendments displayed nearly overlapping trends of ∼60–65% TPH reduction in 90 days, with most of the degradation occurring during the initial 15 days period. Addition of NS also enhanced reduction of TPH with a considerably high efficiency, yielding a maximum reduction of 70%.

**FIGURE 2:**
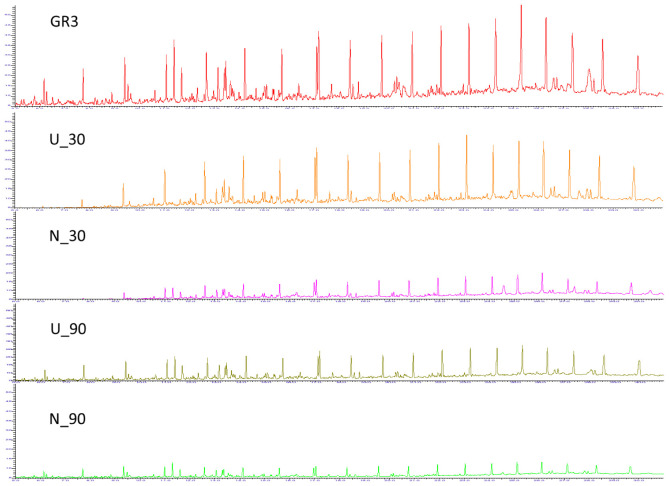
**Gas chromatogram for GR3, U and N samples.** GC-FID chromatograms showing constituent hydrocarbons after 30 and 90 days of microcosm incubation with or without nitrate amendment (U and N). Peaks have been assigned to carbon chain length corresponding to TPH standard (C10–28).

Nature of constituent hydrocarbons within the microcosm derived sludge samples was assessed to gain information on possible role of nutrient amendment in biodegradation of different hydrocarbon compounds by indigenous microorganisms (**Figure 2**). Original GR3 sludge was found to be primarily consisting of medium to long chain length (C12–28, 79%), together with lesser proportions of very long (>C28, 10%) and short chain (<C12, 11%) hydrocarbon compounds. Relative distribution of hydrocarbons (<C12, C12–20, C20–28 and >C28) in the samples with and without N amendment was ascertained during microcosm incubation and normalized percent abundance of each category has been presented in **Table 4** (**Supplementary Figure S4**). Following N amendment, significant (*p* < 0.05) depletion (1.6 fold) of very long chain length compounds (>C28) was observed (compared to its U counterpart showing only 0.3 fold decrease). Medium (C12–20) chain length compounds remained almost unchanged with incubation, while higher chain length (C21–28) hydrocarbons were found to decrease. Proportions of lower chain (<C12) length compounds were increased in both U (single fold) and N (threefold) samples, possibly indicating greater incomplete degradation of higher chain compounds (leading to formation of lower chain hydrocarbons). The isoprenoid:alkane ratios, pristane/n-C17 for U and N amended samples at the end of 90 day incubation was found to be 1.08 and infinity (no nC-17 detected), respectively, while phytane/n-C18 ratio was 0.36 and 0.5 respectively, indicating that compared to U condition, N amendment facilitates considerable biodegradation.

**Table 4:** Carbon chain length distribution in original (0 time), unamended (U) and N amended samples after 30, 60, and 90 days of incubation, quantified through GC-FID.

Carbon chain length distribution	Relative percent abundance of hydrocarbons (%)						
		
	GR3	U_30	U-60	U_90	N_30	N_60	N_90
<C12	10.76	18.39	18.83	22.28	14.72	33.47	42.98
C12-20	35.86	40.29	39.91	38.16	48.23	23.55	41.99
C20-28	42.82	31.95	33.51	32.11	30.34	36.41	11.29
>C28	10.48	9.47	7.62	7.5	7.44	6.39	3.8
*n*-Pristane/C17	0.8506	0.899	ND	1.079	∞	ND	∞


#### Shift in Microbial Community with Amendments

**FIGURE 3:**
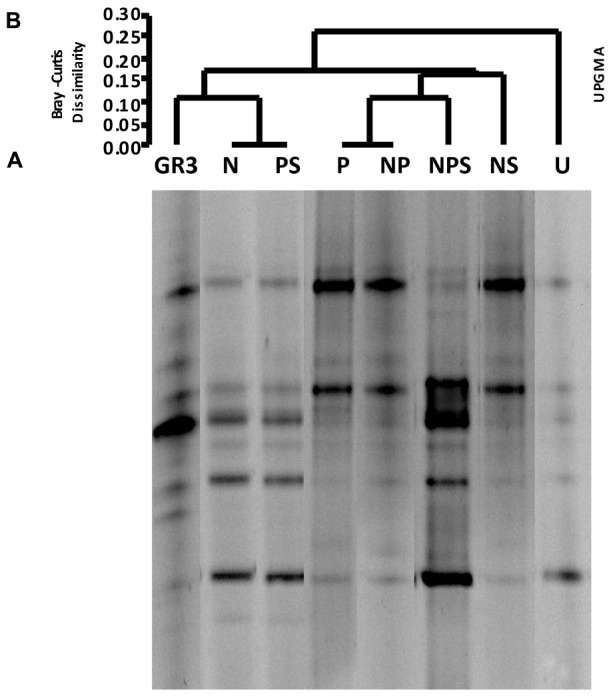
**Denaturing gradient gel electrophoresis of microcosm samples.** DGGE patterns of bacterial communities from microcosms with various amendments **(A)** and Bray–Curtis cluster analysis among the DGGE band patterns **(B)**. No amendment control (U) was also included. For Bray–Curtis analysis presence or absence of bands at corresponding positions was considered.

The effect of aforementioned treatments on microbial community composition was ascertained using DGGE of 16S rRNA gene from the metagenomes extracted after 90 days. Preliminary studies conducted with sample metagenomes in our laboratory showed that compared to V4 region of the 16S rRNA gene, V3 region could provide greater insights (more number of bands with proper resolution) into the bacterial community composition (data not shown) and hence the later was targeted in further study. The DGGE patterns of samples suggested a considerable change in microbial community composition following different treatments as compared to the untreated control (U) as well as the native sample (GR3) (**Figure 3A**). Each distinguishable DGGE band represented a major bacterial population within the community thus reflecting the microbial diversity present in the samples. The number of bands was found to increase in all the treatments over 90 days, supporting the CFU data that suggested the stimulation of bacterial populations to varying degrees upon amendments. Our attempt to find a possible correlation between increase in the number of bands with degradation of hydrocarbons, following different treatments, did not yield any significant result (Pearson coefficient = 0.1598). However, the UPGMA on presence and absence of bands showed a relationship between the treatments (**Figure 3B**). Presence of two distinct clades at a dissimilarity index of 0.15 to 0.20 showed a clustering of band patterns among GR3, N, PS (clade A) and P, NP, NPS, NS (clade B) samples with U cladding separately. The result also indicated a distinct change in community composition following incubation within the microcosm without any treatment (U). Interestingly, even after incubating with N or PS, the community compositions were more similar to the original one, relative to the other treatments (**Figure 3A**).

#### Community Level Physiological Profiling

**FIGURE 4:**
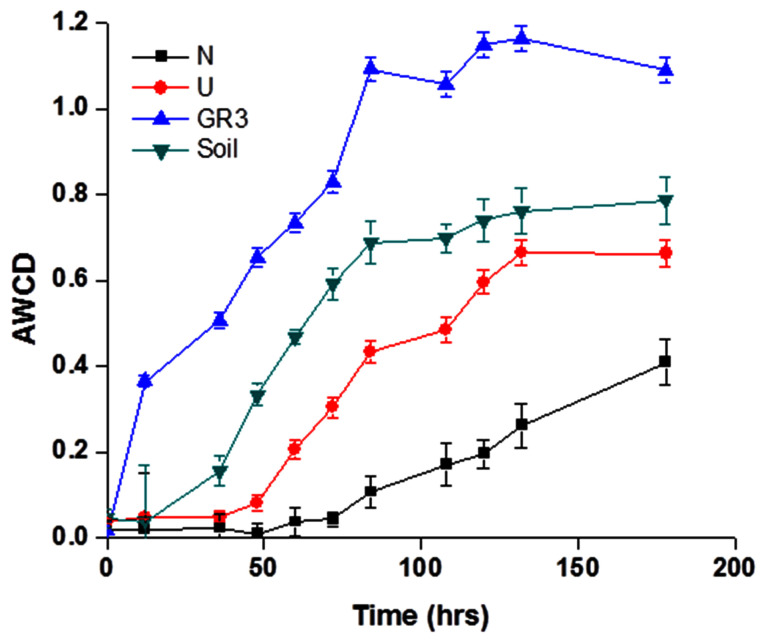
**Community level physiological profile of GR3, U and N samples.** Average well color development (AWCD) plot for four samples, viz., original sludge (GR3), unamended (U) and nitrate amended (N) microcosm samples and non-polluted soil (Soil). Color development was monitored till 7 days. Error bars represent the standard error of mean (*n* = 3).

Metabolic diversity of indigenous populations was ascertained before and after biostimulation (following 90 days incubation) in selected microcosms. BIOLOG Eco plates were used to characterize the community’s physiological profiles. The response of microbial communities’ toward utilization of 31 different carbon sources belonging to six different categories, viz., amino acids, carbohydrates, phenolic compounds, polymers, carboxylic acids, and amines, over a period of 7 days was recorded (**Figure 4**). To understand the characteristic substrate utilization property of the sludge community better, CLPP of an unpolluted soil was also performed. The AWCD data, as obtained from GR3 sample represented rapid and efficient substrate utilization pattern (for all 31 substrates) over the time period, exceeding that of the soil sample significantly. Analysis of utilization profiles for different substrate groups further revealed that members of GR3 had a preference for metabolizing amino acids and carboxylic acids over the rest, while for the soil sample polymers and carbohydrates top the list (**Supplementary Figure S1**). With respect to both N and U microcosms, considerable shift in physiological profiles was noted. A significant reduction in substrate metabolism was shown by the microbial populations in U microcosm which further declined to a much weaker response in N amended populations (**Figure 4**). Although the substrate utilization profile in U microcosm was poor, a preference for carboxylic acid and amino acids was still observed (**Supplementary Figure S1**). N amendment microcosm’s community showed an overall slow utilization pattern but with a distinct preference for the substrates with following order: carbohydrate > polymer > amino acids (**Supplementary Figure S1**). A closer look into the data indicated that none of the 31 different carbon sources were utilized by the N amended community till 75 h, and only fewer substrates were utilized beyond 75 h, indicating possible enrichment of populations with considerably altered metabolic properties.

#### Description of the 16S rRNA Gene Libraries and Community Shift during Biostimulation

Effect of bioremediation treatment (biostimulation) on microbial diversity of the sludge was studied to gain a thorough understanding of the community composition. Deep sequencing of 16S rRNA gene’s V4 region derived from the metagenomes of GR3 sample as well as from two microcosms (N- and NS- amended, which showed enhanced TPH degradation), was performed. The natural shift in indigenous community expected during laboratory incubation was also assessed using the metagenome from microcosm without any nutrient amendment (U) after 90 days. From the four amplicon libraries, a total of 968564 high-quality 16S rRNA gene sequences (average read length∼275 bp) were obtained (**Table 5**). These sequences were assigned into 17381 operational taxonomic units (OTUs) with a 97% similarity limit. Although the rarefaction curves did not reach plateau for some of the samples (**Supplementary Figure S2**), Good’s coverage estimators indicated that the sizes of libraries were sufficient to cover 96–99% of the communities (**Table 5**). Taxonomic identity and compositional characteristics of microbial communities within various microcosms were ascertained in detail.

**Table 5:** Total reads and OTU distribution between four samples obtained through next generation sequencing (Ion-Torrent PGM), diversity indices (calculated using QIIME workflow), taxonomical distribution and functional genes detected (**Table 2**).

Sample_ID	GR3	U	N	NS
Number of reads	577595	45322	149031	196616
OTUs (97% identity)	9449	2584	2696	2652
Estimated total OTUs (Chao1)	18441.06	6323.026	5548.731	4972.729
Shannon evenness index	5.55	5.53	3.79	3.31
Simpson index	0.90	0.91	0.82	0.69
Equitability	0.42	0.49	0.33	0.29
Goods coverage	0.99	0.96	0.99	0.99
Archaeal taxa (% Reads)	17.31	0.23	2.80	5.80
Bacterial taxa (% Reads)	82.03	99.70	97.18	94.20

**Number of archeal taxa detected at**				

Phylum	3	3	2	2
Class	9	5	5	5
Family	14	5	6	10
Genus	13	4	5	10

**Number of bacterial taxa detected at**				

Phylum	45	36	29	34
Class	94	53	42	60
Family	184	95	82	137
Genus	259	105	87	192

**Functional genes detected**				

*alk*B	+	+	+	ND
*bss*A	+	+	+	ND
*mcr*A	+	+	+	ND
*nir*S	+	+	+	ND
*nif*H	+	ND	+	ND
*dsr*B	+	ND	+	ND
*nar*G	+	ND	+	ND
*nrf*A	+	ND	+	ND


*+, detected; ND, Not determined.*

##### Analysis of the GR3 community

Total 156 Mb (10^6^ bases) data was obtained from the GR3 sample representing 577595 quality reads, grouped into 9449 OTUs. Taxonomic affiliation of these OTUs as ascertained by QIIME indicated presence of bacterial (95.3% of reads, 90% OTUs) and archaebacterial (1.86% of reads and 9.7% OTUs) members. A very small number of OTUs (0.3%) corresponding to 2.83% reads remained unassigned. Diversity index indicated Shannon and Simpson indices of 5.55 and 0.9, respectively (**Table 5**). Downstream analysis of OTUs (comparison with using Greengenes database) indicated diverse assemblage of populations viz. 45 phyla distributed in 184 families, represented by 259 genera within the bacterial domain and 3 phyla, 14 families and 13 genera within the archaebacterial domain (**Table 5**).

**FIGURE 5:**
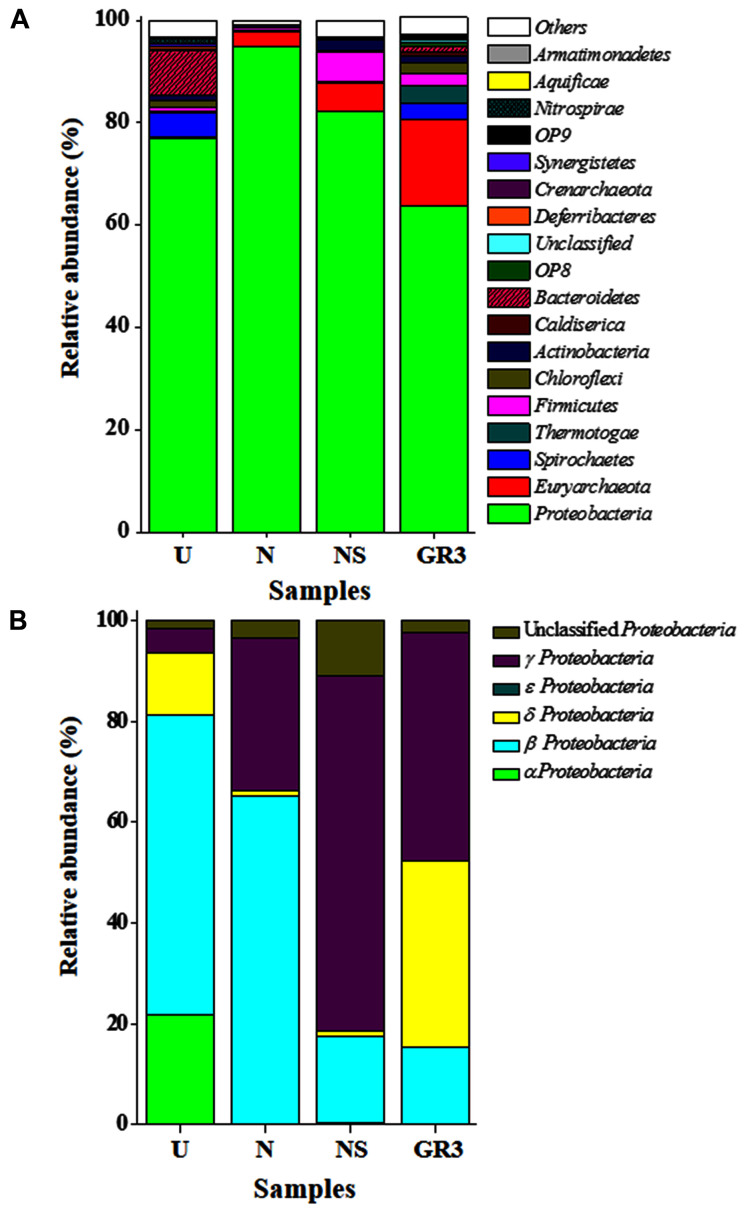
**Distribution of bacterial and archaeal groups in original and microcosm samples (at phylum-class level).** Composition of microbial communities at phylum **(A)** and class (for *Proteobacteria*) **(B)** level. ‘Unclassified’ signifies sequences which did not match any known sequence in the database. OTUs with abundance <0.1% were added up and was represented as ‘Others.’

Among the major (>1% abundance) bacterial and archaebacterial phyla detected, *Proteobacteria* and *Euryarchaeota* together covered 80% (**Figure 5**). *Spirochaetes* (3.2%), *Thermotogae* (3.2%), *Firmicutes* (2.5%), *Chloroflexi* (2.2%), *Actinobacteria* (1.1%), *Caldiserica* (0.97%), *Bacteroidetes* (0.84%), OP8 (0.79%), *Deferribacteres* (0.31%), etc., represented the remaining. Members of 20 candidate divisions and several other bacterial phyla and *Parvarchaeota* (archaebacteria) were detected at very low (<0.3%) abundance. Phylum *Proteobacteria* was composed mainly of γ- (28%), δ- (23.5%) and β- (9.6%) subdivisions along with lesser proportions of α- and ε- *Proteobacteria*. *Gammaproteobacteria* was represented by (Order/Genus) *Xanthomonadales/Pseudoxanthomonas, Stenotrophomonas*, and *Psedomonadales/Pseudomonas, Alkanindiges* and *Acinetobacter*. *Deltaproteobacteria* was represented by *Syntrophobacterales/Syntrophus, Syntrophobacter*; *Desulfuromonadales/Geobacter* and other sulfate reducing genera. The presence of *Rhodocyclales*/*Azovibrio* and *Petrobacter* along with lower proportions of *Azospira, Thauera, Azoarcus* etc. was noted among the β *Proteobacteria*; whereas, *Rhizobiales/Hyphomicrobium*; *Sphingomonadales/Sphingobium; Rhodospirillales/Acidocella* etc. comprised α proteobacterial members.

The second most abundant phylum, *Euryarchaeota*, was represented by hydrogenotrophic, acetate/H_2_/formate oxidizing, methanogenic, hydrocarbon utilizing groups (Order/Genus) *Methanobacteriales/Methanobacterium; Methanosarcinales/Methanosetae*; *Methanomicrobiales* /family *Methanoregulaceae* and genus *Methanolinea* and unclassified *Methanomicrobiales.*

Among the other groups, phylum *Spirochaetae* was represented by *Spirochaetacea*/*Treponema* together with unclassified members; unclassified *Brachyspiraceae* and Sediment 4 of *Leptospirillales.* Anaerobic theromophilic genera *Fervidobacteria* and *Thermotoga* were detected as *Thermotogae* members. Class *Bacilli* and *Clostridia* solely represented *Firmicutes* with presence of diverse genera, including thermophilic, syntrophic/fermentative groups represented by *Coprothermobacter, Sedimentibacter, Thermoanaerobacter, Thermoanaerobacterium, Syntrophomonas*, *etc.*; and *Bacilli/Bacillus, Staphylococcus*, *etc.* Members of the phylum *Chloroflexi* included (Order/Genus) *Anaerolineales/Longilinea, Anaerolinea* etc.; *Dehalococcoidales* and *Thermomicrobium*, while phylum *Actinobacteria* comprised mainly of *Actinomycetales/Mycobacterium, Corynebacterium, Rhodococcus, Dietzia* etc. along with few *Solirubrobacterales* and *Gaiellales* members. Among the other minor phyla detected, *Caldiserica, Bacteroidetes*, candidate division OP8, *Deferribacteres* and *Nitrospirae* (cumulative abundance 3%) garner special attention.

##### Analysis of the biostimulated (N, NS) and Unamended (U) communities

One of the key findings in this microcosm study was the observed change in microbial community composition during the biostimulation based enhanced degradation of petroleum hydrocarbons. As evident from **Table 5**, community richness [Shannon index (*H*)] decreased to 3.79 and 3.31 for N and NS amended microcosms (from a value of 5.55 in GR3), suggesting a selective enrichment of populations following specific amendments during the biodegradation of petroleum hydrocarbons. Unamended microcosm (U) on the other hand retained the community diversity (*H*′ = 5.53) close to the native GR3 sample. Concomitant to the lowering of diversity, numbers of taxonomic groups (phylum, class, family and genus) decreased considerably in each microcosm, with or without any amendments, indicating the possible impact of physiochemical conditions developed within the microcosm (**Table 5**). Number of taxa detected in untreated microcosm (U) were found to be 39 phyla, 58 class, 100 family and 109 genera, which continued to show a significant decline following N and NS amendments, and it was noted to be more drastic with the former (31, 47, 88, and 92), despite its highly positive impact on TPH degradation.

**FIGURE 6:**
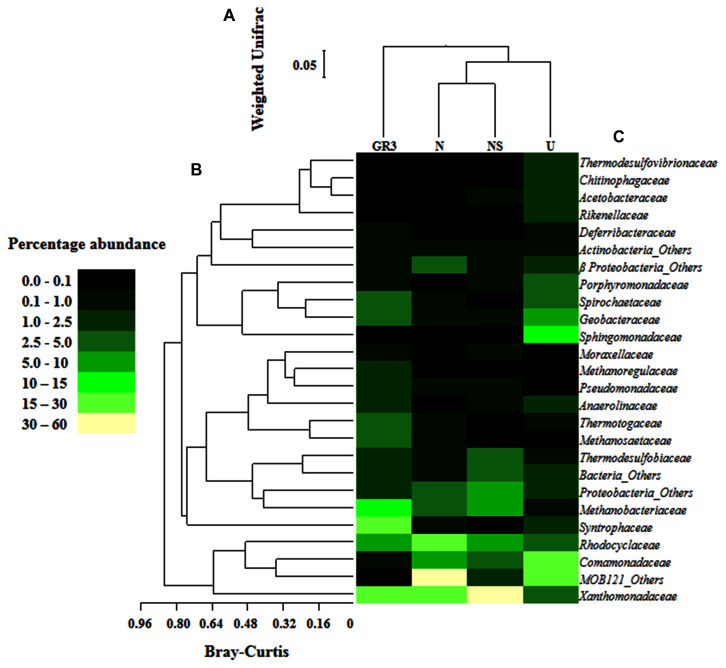
**Relative abundance of major families detected in original and microcosm samples and their interrelations.** Heat map showing relative abundance of major bacterial and archaeal families detected in the four metagenomic libraries **(A)**. Families which gave cumulative relative abundance of >1% have only been considered. Bray–Kurtis relationship **(B)** among the abundance of families across the samples and Weighted UniFrac relationship **(C)** between the samples, based on the composition as represented by the major taxa were also presented. Change in the percent abundance of major taxa at lowest level of identification was presented **(D)**. For determining the change abundance of any specific taxa in microcosm sample was subtracted from the same in GR3.

Microbial community of the U microcosm was found to be dominated by β *Proteobacteria* (mainly unclassified *Comamonadaceae* and unclassified MOB121 members) (46%); α *Proteobacteria* [unclassified *Sphingomonadaceae*, *Acetobacteriaceae* (genera *Sandaracinobacter, Paracoccus, Acidiphilium*, etc.)] (17%) along with *Bacteroidetes* (unclassified *Chitinophagaceae, Rikenellaceae*) (8.5%), *Spirochates* (*Treponema* etc.) (5%), δ (*Geobacter*, *Syntrophus*) and γ *Proteobacteria* (*Pseudoxanthomonas*) (4%) (**Figure 5**). Members of *Chloroflexi* (1.5%), *Nitrospirae* (1.2%), *Actinobacteria* (0.9%), *Firmicutes* (0.8%) and *Synergistetes* (0.3%) were also present in low abundance. Comparison between GR3 and U communities indicated a decrease in frequency of reads affiliated to various groups of *Archaebacteria*, δ- and γ- *Proteobacteria, Thermotogae, Firmicutes, Actinobacteria* etc. (**Figures 5** and **6**). In this regard, a noticeable finding was the loss of anaerobic, acetotrophic, sulfate/nitrate/iron reducing, syntrophic members like *Xanthomonadaceae* (*Pseudoxanthomonas*), *Syntrophaceae* (*Syntrophus*); archaebacterial taxa *Methanobacteriaceae* (*Methanobacterium*) and *Methanosaetaceae* (*Methanosaeta*); *Thermodesulfobiaceae* (*Coprothermobacterium*), *Thermotogae* (*Fervidobacterium*). In contrast to this, an increase in abundance of facultative photoorganotrophic/fermentative, storage lipid (polyhydroxybutyrate) degrading, N_2_ fixing/ammonia oxidizing bacterial taxa namely *Sphingomonadaceae*, *Comamonadaceae*, unclassified group of β *Proteobacteria*, *Geobacteriaceae*, anaerobic coprophagous bacteria of *Porphyromonadaceae*, etc. was observed.

Following nitrate amendment the community showed a drastic change in its composition (**Figure 6**). It was observed to be predominated by β- (unclassified MOB121, *Comamonadaceae* members *Azovibrio*) (62%) and γ- (*Pseudoxanthomonas*) (29%) *Proteobacteria;* along with unclassified *Proteobacteria* (3.4%), *Archaebacteria* (3%) [*Methanobacteria* (*Methanobacterium*), *Methanomicrobia* (*Methanosaeta*)] and *δ Proteobacteria* (*Geobacter*) (1%). Lower proportions of members already detected in GR3, viz., *Firmicutes* (*Coprothermobacter* and *Bacillus*) (0.6%), *Actinobacteria* (*Rhodococcus*), *Thermotogae* (*Fervidobacterium*), *Spirochetes* (*Treponema*) (0.1%) etc., were also detected in this sample. Comparison of the N community with that of GR3 and U revealed that nitrate addition possibly allowed the community to regain its native composition at least with respect to some of the characteristic groups indicating an extent of community resilience. Particularly, the predominance of nitrate reducing and N_2_ fixing genera *Pseudoxanthomonas* and *Azovibrio*, respectively, together with methane metabolizing *Methanosaeta* and *Methanobacterium* was noticeable. Relative abundance of few other members (unclassified β *Proteobacteria* and *Comamonadaceae; Thermodesulfobiaceae; Thermotogaceae*, etc.), however, resembled that of the U community, perhaps demonstrating the effect of incubation condition within the microcosm.

**FIGURE 7:**
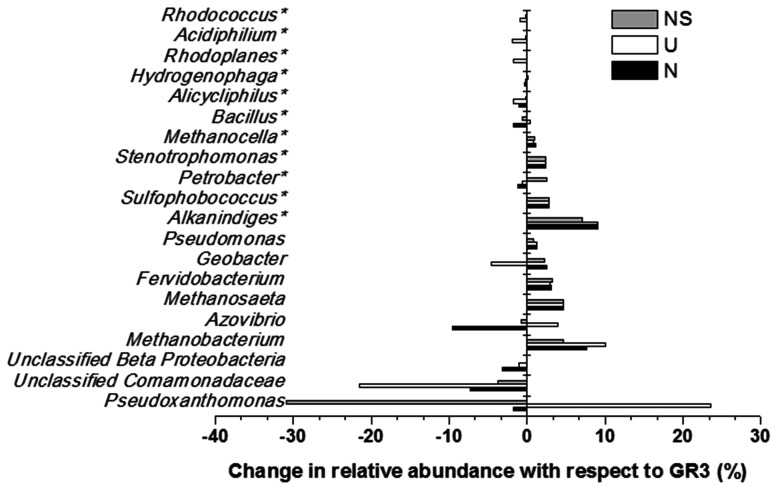
**Effect of amendments on microbial community composition (at genus level).** Change in percentage abundance of genera in microcosm samples U, N and NS, as compared to original sludge, GR3. The abundance for genera marked with asterisk (^∗^) have been multiplied by a factor of 10 to indicate the change prominently. Negative values indicate increase and positive values indicate decrease in percentage abundance with respect to GR3.

Microbial diversity in microcosm with both nitrate and surfactant amendment (NS) was consistent with that of N microcosm, distinctly with respect to the preponderance of *γ Proteobacteria* (mainly, *Pseudoxanthomonas* and *Pseudomonas, Alkanindiges* as minor groups) (58%) along with relatively lesser proportions of β *Proteobacteria* (*Comamonadaceae*, *Azovibrio*) (14%), unclassified *Proteobacteria* (9%), *Firmicutes* (*Coprothermobacter*) (6%), *Euryarchaaeota* (*Methanobacteria*) (6%) and *Actinobacteria* (OPB41) (2%). All the above data have been summarized in the form of a heat-map (**Figure 6A**) based on normalized abundance of 26 most abundant families, sum of abundance of which in all four samples was at least 1%. Bray–Curtis dissimilarity analysis was used to quantify the compositional changes during different treatments (**Figure 6B**). Among the four libraries, most abundant taxa (families) were used to delineate the correlation among them (**Figure 6C**). A number of families showed significant correlation with respect to their relative abundance during treatments. Change in abundance of *Comamonadaceae* and MOB121 of β *Proteobacteria* correlated very well along with *Rhodocyclaceae* and *Xanthomonadaceae*, thus indicating a close agreement between the relative abundance of these groups. Similarly, *Thermotogaceae* and *Methanosaetaceae*, the thermophilic hydrogen producing and methanogenic groups; fermentative and anaerobic metal reducing *Spirochaetaceae* and *Geobacteriaceae*; *Chitinophagaceae*, *Acetobacteriaceae*, and *Thermodesulfovibrionaceae* showed strong coherence in their abundance. The relationship among the samples was further analyzed using Weighted UniFrac analysis which substantiated the fact that the microbial community of original sludge (GR3) was significantly different from any of the microcosm samples. The change in abundance of bacterial and archeal taxa at genera level, in N, U and NS microcosm samples, with respect to GR3, have been presented in **Figure 7**. In **Figure 7** negative values indicate increase and positive values decrease in percentage abundance of respective genera, as compared to GR3. Change in diversity of prokaryotes in microcosm samples could not be discussed thoroughly at genera level owing to the fact that only 29 and 50% of quality reads could be classified at this level in U and N microcosm samples, whereas, in case of NS and GR3, it represented 80 and 85% of reads. However, increase in genera *Azovibrio*, unclassified *Comamonadaceae* and *Pseudoxanthomonas*, was prominent in N, while *Pseudoxanthomonas* alone was found to predominate NS sample.

#### Characterization of Functional Genes

**FIGURE 8:**
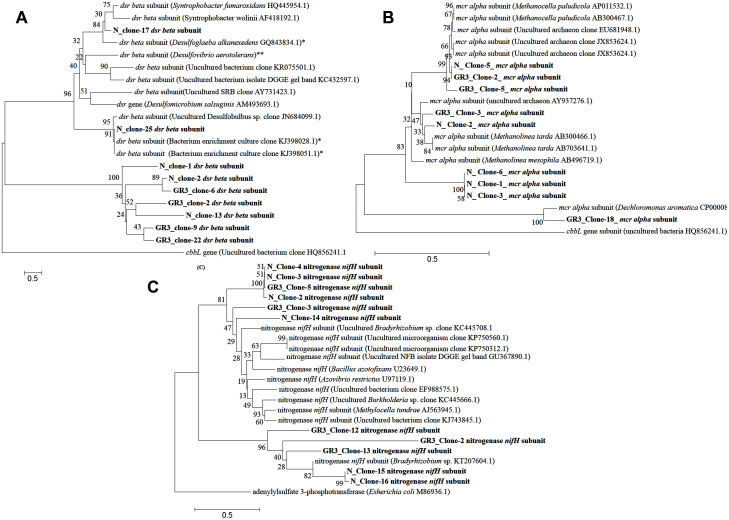
**Phylogenetic distribution of functional genes in GR3 and N.** Neighbor-joining analysis was performed with 1000 bootstrap repeats to generate each tree using MEGA 6 software. Phylogenetic tree for **(A)** *dsr*B, **(B)** *mcr*A and **(C)** *nif*H gene segments from GR3 and N samples. Trees were constructed nucleotide sequence of each gene along with their closest BLASTN matches.

In order to gain an insight into the catabolic potential of the sludge community in terms of biodegradation of hydrocarbons and relevant metabolic pathway, a set of functional genes was targeted. Genes related to hydrocarbon degradation (*bss*A and *alk*B), N_2_ fixation (*nif*H), nitrate reduction (*nar*G and *nir*S), methane metabolism (*mcr*A) and sulfate reduction (*dsr*B) were analyzed. All the genes could be detected in original sludge (GR3) and sludge incubated with nitrate (N). Among these genes, *bss*A, *alk*B, *mcr*A, *dsr*B, *nar*G and *nif*H were cloned and sequenced for their analysis. Five to ten clones for each gene was sequenced to identify their closest match (**Supplementary Table S1**). The gene involved in methane metabolism, alpha subunit of methyl CoM reductase (*mcr*A), could be detected and their sequences matched to similar gene of methanomicrobial genera *Methanolinea, Methanobacteria* or *uncultured archaeon* (**Figure 8A**). Dissimilatory sulfite reductase gene (*dsr*B) showed close similarity to *dsr*B gene from *Peptococcaceae* bacteria (*Firmicutes*) and uncultured sulfate reducing bacterium (**Figure 8B**). Gene encoding a fragment of nitrogenase enzyme responsible for N_2_ fixation (*nif*H*)* was present in the sludge community and clones showed close similarity with the same gene from archaeal origin namely *Methanosarcinae, Methanocella, Methanolinea*, etc. (**Figure 8C**). The nitrate reductase (*nar*G) gene associated with nitrate reduction, although detected, but its clones did not show similarity to the same gene. However, the *nar*G sequences showed close proximity to thioredoxin reductase protein family present in β proteobacterial genus *Azovibrio*. Two hydrocarbon metabolizing genes *alk*B and *bss*A did not show significant similarity to known proteins. While *alk*B sequence showed match with ATPase protein family member of *Methanobacterium, bss*A showed match to hypothetical proteins (**Supplementary Table S1**). This data further substantiated the fact that denitrifying, methanogenic, hydrocarbonoclastic bacterial community present in the original sludge is being maintained and functioning in presence of nitrate amendment and anaerobic pockets might have been produced in the sludge microcosm owing to functioning of anaerobic bacterial as well archaebacterial community. Physicochemical monitoring of the microcosm indicated lowering in DO and ORP following N amendment (data not shown) thus possibly corroborating the observed changes in microbial catabolism/ taxonomic characters.

### Biostimulation of Hydrocarbon Degradation by N Amendment with Other Oily Sludge

**FIGURE 9:**
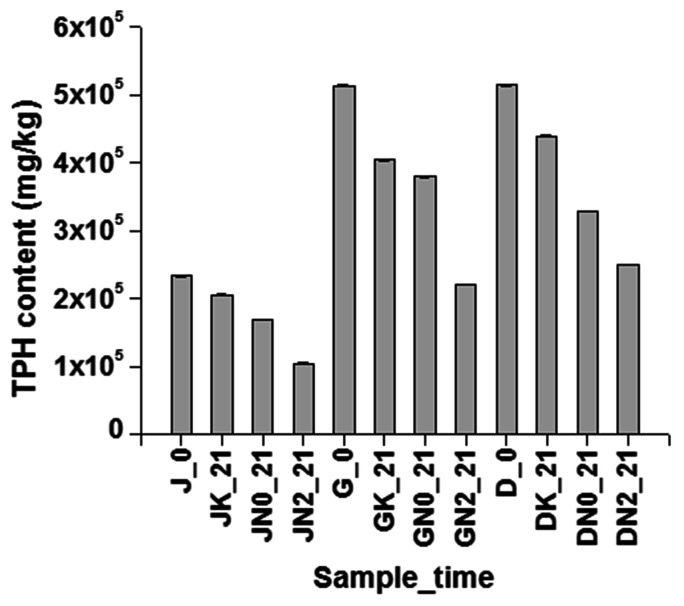
**Effectiveness of nitrate amendment in other similar samples.** Effect of nitrate amendment on hydrocarbon degradation on petroleum sludge collected from Duliajan (J), Digboi (D) and Guwahati Refinery (G), at 0 day and after 21 days incubation. K signifies sodium azide treated sample, abiotic control, N0 signifies unamended sample, natural attenuation and N1 signifies biostimulated samples with 10 mM NaNO_3_. TPH have been measured using GC-FID.

In order to verify the effect of nitrate addition on the degradation of hydrocarbon compounds, two more sludge samples collected from other refineries in the North-East region of India, were also tested through similar nitrate amendment based approach. As evident from **Figure 9**, TPH degradation was markedly enhanced in all the three sludge samples, with nitrate amendment as opposed to U as well as abiotic controls. In all the cases abiotic losses amounted to 10–20% TPH depletion, while in U controls TPH degradation varied from 26 to 35%. Nitrate amendment (either in low or in high concentrations) resulted in considerable degradation of hydrocarbons (30–55%) within the incubation time of 21 days. This microcosm further validated the effect of nitrate in stimulating indigenous microbial population toward enhanced petroleum hydrocarbon degradation.

## Discussion

Understanding the microbial community composition, structure and dynamics within contaminated niche is critical for designing *in situ* bioremediation strategies. In this study, we have analyzed the prevalent geochemical and microbiological environment of the refinery waste and evaluated the scope for enhanced bioremediation using selected amendments. Together with anoxic and reducing environment, high petroleum oil content resulting in deficiency of other nutrients (N,P) and presence of various heavy metals designate this sludge as a distinct ecosystem for the inhabitant microorganisms. The variety of chemical components including N-, S-, hydrocarbons, heavy metals that coexist within the oil contaminant niche can be utilized by the inhabitant organisms as electron donors or acceptors or as other nutrient resources. Biogeochemical processes that prevail within the hydrocarbon rich petroleum sludge largely depend on the microbial community, nature and quantities of available nutrients and microbial metabolic potential. In this study we observed a slow rate of natural attenuation (as evident from high pristane/nC17 ratio) owing to the paucity of adequate N and other alternate electron acceptors. Slower rate of intrinsic biodegradation of constituent hydrocarbons in various HREs has been attributed to lack of available electron acceptors and/or inadequate N availability (Fathepure, 2014; Head et al., 2014; Smith et al., 2015 and references therein). Unlike the other electron acceptors, sulfate, which is generally present at elevated level in many HREs is thermodynamically less resourceful compared to nitrate and nitrite and hence oxidative metabolism utilizing sulfate under anoxic condition may not result in appreciable biodegradation of hydrocarbons. Considering these limiting factors, biostimulation of hydrocarbon degradation by indigenous organisms with selective nutrient addition has been recommended for developing *in situ* bioremediation strategy (Röling et al., 2002; Margesin et al., 2007; Wang et al., 2012; Dashti et al., 2015; Zhang and Lo, 2015).

Metagenome derived 16S rRNA gene amplicon sequencing could successfully resolve the first question we posed in this study delineating the composition of autochthonous microbial community within oil refinery waste sludge. Noticeably, the N limited, hydrocarbon rich sludge showed presence of a diverse and metabolically versatile community with a set of ‘core anaerobic population’ coexisting with aerobic and facultative anaerobic organisms. Within the waste storage tank the upper surface layer remained continuously exposed to air allowing obligate aerobes to prevail and function therein. Lack of mixing and exposure to air, coupled with low diffusion of O_2_ through the heavily oil rich interior of the stored sludge, led to the formation of anoxic and anaerobic zones within and toward the bottom of the oil sludge. While the ‘core anaerobic populations’ could potentially drive syntrophic-methanogenic metabolisms in highly O_2_ limiting, reduced zones, organisms capable of surviving under aerobic-facultative anaerobic conditions thrive well within the relatively O_2_ available zones and thus constitute another functionally active part of the community (An et al., 2013).

Major players of anaerobic populations constituted of δ *Proteobacteria* and methanogenic (acetotrophic/hydrogenotrophic) *Euryarchaeota* (archaebacteria). Co-occurrence of hydrogenotrophic, acetotrophic and methylotrophic organisms could be implicated toward the ‘methanogenic hydrocarbon-associated’ biodegradation property of the community (Tan et al., 2015). Methanogenic hydrocarbon metabolism and biodegradation have been reported as an important biogeochemical process elsewhere (e.g., natural gas deposits, etc.) but not in refinery/oil processing industry wastes (Jones et al., 2008; Kleinsteuber et al., 2012; Gieg et al., 2014; Tan et al., 2015). Under anaerobic condition the interplay of syntrophic bacteria and methanogenic archaebacteria facilitates degradation of hydrocarbons. Syntrophic organisms (*Syntrophaceae, Geobacteraceae*, etc.) catalyze the degradation of hydrocarbon substrates to produce H_2_ and/or acetate, which are subsequently utilized by methanogens (*Methanobacteriaceae, Methanoregulaceae, Methanosaetaceae*) (Gieg et al., 2014; Tan et al., 2015). Members of the families *Syntrophaceae* and *Geobacteraceae* have also been previously reported to be capable of long chain alkane degradation utilizing metals as TEAs. Other anaerobic taxa detected include members of *Firmicutes*, primarily affiliated to obligatory anaerobic genus *Coprothermobacter.* Member of this genus is previously implicated in monoaromatic hydrocarbon degradation along with δ *Proteobacteria* (Fowler et al., 2014). Among the other taxa, *Fervidobacterium*, *Decholoromonas* and methylotrophic *Methyloversatilis* are generally present as part of anaerobic methanogenic community involved in aerobic/anaerobic benzene metabolism (Coates et al., 2001).

The observed predominance of γ *Proteobacteria* in refinery sludge is in agreement with several other HREs including natural oil deposits, asphalt, crude oil, oil sand, oil contaminated water, soil and sludge, etc. (Militon et al., 2010; Yergeau et al., 2012; Benedek et al., 2013; Silva et al., 2013; Aleer et al., 2014; Das and Kazy, 2014; Lamendella et al., 2014; Head et al., 2014; Yang et al., 2014; Smith et al., 2015). Members of genus *Pseudoxanthomonas (γ Proteobacteria), Azovibrio* (β *Proteobacteria*) and several others (*Pseudomonas*, *Alkanindiges*, *Stenotrophomonas, Petrobacter*, *Azoarcus*, *Thauera* detected at higher proportions in GR3 sample represented the aerobic-facultative anaerobic cluster. *Pseudoxanthomonas* is known as a hydrocarbon degrading and nitrate/nitrite reducing organism that often occupy moderately thermophilic habitats (Reinhold-Hurek and Hurek, 2006; Alonso-Gutiérrez et al., 2009; Patel et al., 2012; Guazzaroni et al., 2013). Together with this group, presence of aerobic/microaerophilic, N_2_ fixing *Azovibrio*, capable of using oxygen, nitrate or even perchlorate as TEA (Reinhold-Hurek and Hurek, 2006), conformed to the robust metabolic architecture of the community. Abundance of *Pseudoxanthomonas* and *Azovibrio* together with anaerobic, methane producing taxa is a less observed phenomenon; although presence of aerobic organisms in highly anoxic oil-rich environment is not rare. Aerobic bacteria could possibly represent the members of the ‘cryptic aerobic community’ utilizing oxygen of the air or produced within the sludge through chemical/biochemical transformations or even represent aerobes who could function anaerobically as well (Head et al., 2014).

Functional potential of the constituent populations was further validated by the presence of nitrate/nitrite reduction (*nar*G, *nir*S), dissimilatory sulfate reduction (*dsr*B), methane metabolism (*mcr*A), anaerobic hydrocarbon activation (*bss*A) as well as aerobic alkane degradation (*alk*B) related genes from the metagenome. Degradation of *n*-alkane under aerobic condition is catalyzed by hydroxylation (at terminal or sub-terminal methyl group) through hydroxylase [methane monooxygenases (MMOs), cytochrome P 450 monooxygenases (pMMOs), alkane and alkane like hydroxylase (*alk*B and *alk*B like)] (Rojo, 2009; Kleinsteuber et al., 2012; Fuentes et al., 2014; Sierra-García et al., 2014). Catabolism of hydrocarbons under anaerobic condition is facilitated by using fumarate adding succinyl synthase (encoded by *bss*A like genes, i.e., *bss*A, *ass*A and *nms*A) gene encoding enzymes that are found to be widespread among nitrate, sulfate, iron reducing, methanogenic and fermentative microbial members covering broad range of phyla, from *Firmicutes* to *Proteobacteria* (Kniemeyer et al., 2007; Grossi et al., 2008; Jones et al., 2008; Callaghan et al., 2009; Higashioka et al., 2009; Kleinsteuber et al., 2012). Dissimilatory reduction of nitrate to N_2_ or intermediate products are catalyzed by several enzymes working in tandem, some of which are nitrite reductase (*nir*S) and nitrate reductase (*nar*G), while N_2_ fixation is normally catalyzed by nitrogenase enzyme whose iron subunit is encoded by the *nif*H gene.

Bioremediation of petroleum hydrocarbon contaminated wastes through either bioaugmentation or biostimulation or both have been well studied, although remediation of high TPH containing wastes is relatively less. We have compiled the published reports on bioremediation attempts made to reduce the TPH load in various wastes and found that even a waste sludge containing 372 g TPH/kg could be treated to the extent of 95% TPH reduction (**Table 6**). During our study, we have observed a distinct superiority of nitrate in improving the intrinsic hydrocarbon biodegradation potential of the community. Addition of N substrates to nutrient deficient hydrocarbon-rich samples has been reported to increase cell growth rate and increase the rate of hydrocarbon degradation (Walworth et al., 2007). Among the various N compounds tested, nitrate, ammonia and urea are commonly used. Addition of fertilizers or urea has been shown to have negative impact on microbial populations due to ammonia overdosing and/or acid toxicity, which significantly inhibited biodegradation (Stroud et al., 2007; Bell et al., 2013a,b; Dashti et al., 2015). On the other hand, nitrate addition favorably alleviates the N deficient state of high organic carbon-rich sludge and helps in stimulating a plethora of biogeochemical transformations (Mason et al., 2014; Zhang et al., 2015). Added nitrate have been known to play key role as TEA to microorganisms in an oxygen deficient environment. Being more thermodynamically favorable and taking part in assimilatory and/or dissimilatory reduction process, it facilitates heterotrophic or autotrophic denitrification, coupled to simultaneous oxidation of organic matter. Under oxygen limiting and anaerobic conditions, apart from being used as TEA, nitrate can also act toward activating the hydrocarbons for their biodegradation (alkanes, in particular) (Zedelius et al., 2011). In the present study, increase in CFU counts and TPH degradation showed good agreement with nitrate addition as both were favored maximally during the first 15 days, followed by near saturation of cell numbers till 60 days corroborating several other reports (Margesin et al., 2007; Machin-Ramirez et al., 2008; Mair et al., 2013). The level of biodegradation achieved by nitrate amendment in our sample (80% in 12 weeks) corroborated well with earlier reports on biostimulation studies (Bento et al., 2005; Margesin et al., 2007; Machin-Ramirez et al., 2008; Couto et al., 2010; Mair et al., 2013; Jasmine and Mukherji, 2015). GC-FID based analysis of C chain length distribution showed that higher C chain length compounds could be efficiently degraded following N amendment. The depletion of >C28 hydrocarbon compounds with N amendment indicates that these compounds could either be completely degraded to CO_2_ or partially degraded to form medium to high chain length compounds, the percentage of which were observed to increase collaterally. This provides a clear indication of utilization of higher C length hydrocarbons in microcosm samples, either completely or partially, and is significantly enhanced in the presence of nitrate. Pristane and phytane, the isoprenoid compounds present in the oily sludge have been used as biomarkers owing to their lower degradation potential. The rate of degradation of these isoprenoids compared to corresponding length *n*-alkanes provides useful insights into the extent of biodegradation and microbial activity in hydrocarbon rich sample (Jasmine and Mukherji, 2015). During this study pristane:nC-17 ratio indicated enhanced biodegradation with nitrate amendment, however, preferential degradation of longer chain compounds as detected seemed to be an unusual or less observed phenomenon (Smith et al., 2015). It is generally observed that during biodegradation shorter chain alkanes are preferred over their longer counterpart mostly due to poor solubility of the later and lack of proper metabolic machinery for the same (Sanni et al., 2015).

**Table 6:** Comparison of bioremediation performance for very high TPH content oily sludges (including our sample).

Sl No	Initial TPH concentration (g kg^-1^)	Nature of contamination	Remediation approach	Percentage TPH reduction (%)	Reference
1	400	Refinery sludge from waste lagoon	Biostimulation	>80% within 90 days	This study
2	160.30 – 372.50	Refinery oily sludge	Bioaugmentation with microbial consortia	Upto 95% within 2–12 months	Mandal et al., 2012
3	370	Refinery sludge	Bioaugmentation and composting	Upto 46–53% degradation within 56 days	Ouyang et al., 2005
4	334	Oil sludge from a natural gas processing facility	Biostimulation by balancing C:N:P ratio using inorganic nutrients	Upto 32–51% after 30 days with C/N/P ratio of 100/1.74/0.5.	Roldán-Carrillo et al., 2012
5	110	Oil tank bottom sludge	Combined biostimulation and bioaugmentation (fungal-nutrient amendment)	Upto 91% degradation	Adetutu et al., 2015
6	300	Contaminated soil from refinery	Biopile biostimulation	60% degradation in 3 months	Marín et al., 2006
7	220	Bottom sludge of oil separating tank	Bioaugmentation (*Bacillus subtilis*, *Bacillus megaterium*, *Achromobacter xylosoxidans*, *Pseudomonas fluorescens*, *Candida tropicalis* and *Rhodotorula dairenensis)*	Upto 80.6% within 1-year	He et al., 2014
8	130	Weathered oily waste (PB401) from a 10 years old disposal site	Slurry based bioaugmentation and biostimulation	Upto 24% biostimulation	Machin-Ramirez et al., 2008,
9	99.2	Artficial contamination with Barauni refinery sludge, Bihar, India	Bioaugmentation [*Acinetobacter baumannii* (S19, S26, S30), *Burkholderia cepacia* (P20), *Pseudomonas* sp. (S24)] using carrier based bacterial consortium and nutrients	Upto 90.2% within 120 days	Mishra et al., 2001
10	250	Oil sludge contaminated soil	Biostimulation using manure	Upto 58.2% in 360 days	Liu et al., 2010
11	15–80	Oily sludge	Biostimulation with inorganic nutrients	Upto 70–90% during 2 months	Admon et al., 2001
12	22–55	Sediment samples from oil contaminated land	Biostimulation and bioaugmentation	C14–23, 46–67% in presence of inorganic nutrients with or without bacteria within 90 days	Abed et al., 2014
13	25	Crude oil contaminate soil samples	Microcosms with artificially contaminated sample with biostimulation and bioaugmentation techniques	80% with combined biostimulation and bioaugmentation in 28 weeks	Roy et al., 2014
14	14	Contaminated soil sample from oil storage site	Combined bioaugmentation and bioaugmentation (consortia: *Gordonia alkanivorans* CC JG39, *Rhodococcus erythropolis* CC-BC11, *Acinetobacter juni*i CC-FH2, *Exiguobacterium aurantiacum* CC-LSH-4 and *Serratia marcescens* KH1)	Upto 80% (reduced from 14 to 2) within 140 days	Liu et al., 2011
15	13	Diesel contaminated site	Biopile with the aid of biostimulation	Upto 85% in 76 days	Chemlal et al., 2013
16	10.4	Industrial site contaminated by progressive leakage	Oxygen biostimulation in column fixed bed reactor	Upto C12–20, 80% C21–35 38%, C35–40 44%. within 2 y	Militon et al., 2010


In order to reason the predominance of anaerobic realm of the sludge, and its physicochemical status following bioremediation, several parameters like pH, ORP, DO, and conductivity were assessed in the beginning and at the end of microcosm incubation (**Supplementary Figure S3**). The oxidation-reduction potential (ORP), which indicates the status of the electron transfer regime in the contaminated sample, showed presence of a reducing environment (-50 mV) initially, which decreased with nitrate addition (-150 mV). Simultaneously, pH was found to be alkaline (pH 9) in the beginning which reduced to normal pH in U and slightly acidic (pH 5.8) in N amended samples. Dissolve oxygen (DO) level showed a sharp decline in from 2.24 mg L^-1^ to 0.63 mg L^-1^. The observed change in measured parameters clearly indicates enhanced hydrocarbon oxidation in presence of added nitrate. With elevated biodegradation, dissolve oxygen gets consumed while facultative anaerobes result in to the formation of acetate and other organic acids through fermentation leading to concomitant lowering of DO and pH (Li and Bishop, 2004).

The effect of specific biostimulation strategy (nitrate amendment) on indigenous microbial community composition and their metabolic activities was vividly documented during this study. Analysis of the microbial community dynamics following biostimulation trials using DGGE revealed that except the U set, number and intensity of bands in all treatments changed significantly, indicating selection and enrichment of specific groups following the amendments. Since the respective DGGE bands could not be sequenced, we were unable to delineate the specific group(s) responsible for each band. Although our 16S rRNA gene amplicon sequencing data explained the extent of variation in community composition, enrichment of microbial population with altered metabolic characteristics was also evident from the CLPP data. Following nitrate amendment, the observed sharp change in metabolic abilities of the population could be considered as an indicator of the enrichment of more specialized groups, is in line with previous report. A similar trend of suppression of metabolic activities during petroleum bioremediation with urea addition was reported by Wang et al. (2012).

Metagenome based 16S rRNA gene survey further elaborated the effect of nitrate amendment on microbial community composition during TPH biodegradation highlighting the distinct roles of N metabolizing (nitrate reducing and N_2_ fixing) groups. Since, N cycling is almost exclusively mediated by microbes; change in the community’s composition and biodegradation ability following nitrate addition clearly indicated the possible interplay of organisms involved in simultaneous hydrocarbon degradation and N metabolism. Most of the populations, either stimulated or maintained after nitrate amendment are affiliated to β- and to some extent γ- *Proteobacteria*, as well as methanogenic or methylotrophic archaeal groups. Concomitant increase of *Pseudoxanthomonas* and *Azovibrio* along with unclassified β *Proteobacteria* in response to nitrate amendment indicated probable interplay of these groups in the dynamics of enhanced hydrocarbon degradation. Increased abundance of *Pseudoxanthomonas* is very significant highlighting its role in enhanced biodegradation in presence of nitrate. Members of this genus are known for their nitrate/nitrite reducing, hydrocarbon metabolizing properties and found in various hydrocarbon contaminated environments (Alonso-Gutiérrez et al., 2009; Patel et al., 2012; Guazzaroni et al., 2013). A recent study on *Pseudoxanthomonas* genome analysis revealed the details of genomic inventories of such organism for hydrocarbons (BTEX) metabolism (Choi et al., 2013). This increase in nitrate reducing pseudoxanthomad population following nitrate amendment is in good agreement with observed lowering of DO and ORP of the N microcosm further confirming the possible role of such organisms in hydrocarbon biodegradation. Since we have not created any obligatory anaerobic environment within the microcosm vials, denitrifying bacteria present therein start utilizing the added nitrate as soon as the dissolve oxygen is consumed by obligatory aerobic members. Most of the denitrifying organisms are versatile and can use both O_2_ and nitrate as terminal electron (Chen and Strous, 2013). Increase in abundance of another two groups, *Comamonadaceae and Rhodocyclaceae*, also known to play key roles in N cycle (N_2_ fixation/ammonium oxidation/nitrate reduction) during N amended condition further corroborates our hypothesis on promotion of hydrocarbon metabolism by nitrate amendment. Although no previous reports can be found on the utilization of hydrocarbon substrates by microaerophilic N_2_ fixing *Azovibrio*, members of this genus can use O_2_, nitrate, or even perchlorate as TEA (Reinhold-Hurek and Hurek, 2006). Moreover, diazotrophy was reported to be common in hydrocarbonoclastic bacteria and they may directly or indirectly play an essential role in N metabolism as well as hydrocarbon degradation (Dashti et al., 2015).

**FIGURE 10:**
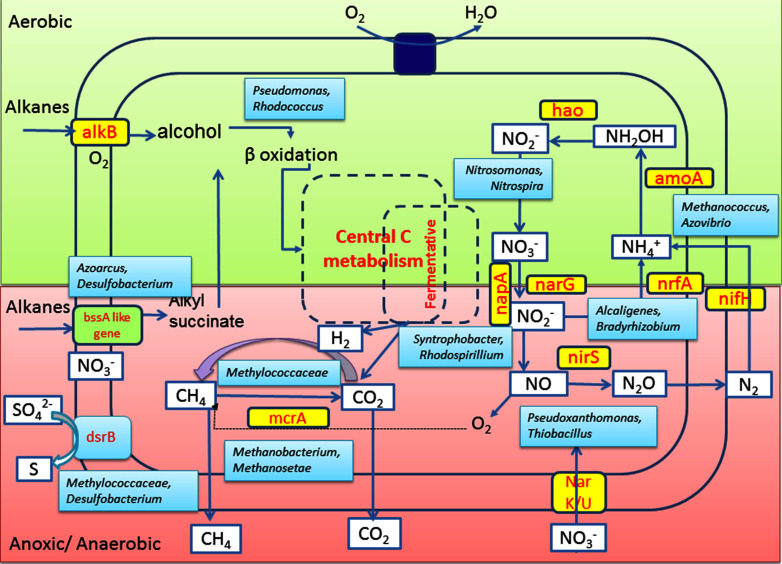
**Conceptual model depicting the interplay of microbial groups in hydrocarbon metabolism and nitrogen cycling in the oil sludge sample GR3.** Microbial community’s potential toward alkane hydrocarbon metabolism and nitrogen metabolism under aerobic (light green) and anaerobic (light pink) conditions are hypothesized based on key genes of various processes or organisms known to be responsible for such processes as detected in this study.

Together with notable presence of these N metabolizing taxa, detection of relevant genes for assimilatory or dissimilatory nitrate reduction (viz., *nar*G, *nrf*A and *nir*S) and N_2_ fixation (*nif*H) in GR3 as well as in N amended sludge indicated the presence of genomic repertoire in the community. Biostimulation of hydrocarbon constituents by nutrient amendment required the involvement of both hydrocarbon metabolizing enzymes as well as enzymes essential to utilize the available nutrients. The presence of genes *nif*H, *nir*S and *nar*G within the N and NS amended sets confirmed their possible involvement in N metabolism. Together with these genes, presence of *mcr*A, *bss*A and *alk*B in all the treatments confirmed that the indigenous populations encoding such determinants are relatively more abundant, particularly within the N treated microcosm that showed highest hydrocarbon biodegradation. Detection of *mcr*A gene encoding α-subunit of enzyme involved in final methane formation step in methanogenesis (Das and Kazy, 2014 and references there in) further corroborated with the methanogenic hydrocarbon degrading population of sludge samples. A summarized overview of the results allowed us to hypothesize a probable conceptual model for better understanding of the microbial metabolic processes in the refinery waste sludge (**Figure 10**). Metagenomic diversity indicated presence of a metabolically rich community in the sample supported by the required genetic repertoire for biodegradation of petroleum hydrocarbons. The model has been developed considering alkanes as a model contaminant and based on the genes detected in the metagenome. Detection of genes related to hydrocarbon degradation (*bss*A, *alk*B), N metabolism (*nif*H, *nar*G, *nir*S), methane metabolism (*mcr*A) and sulfate reduction (*dsr*B), as well as organisms known for harboring such key genetic determinants (critical for metabolism of hydrocarbons) is in good agreement highlighting the community’s potential for hydrocarbon degradation.

The specific community composition may depend on constituent hydrocarbon structure, nutritional and physicochemical conditions prevailing on-site. The insights into the metabolic interplay among the oil sludge community with nitrate amendment will help us in better understanding the dynamics of degradation as well as in development of bioremediation technology. While the metagenomic survey explored the microbial community structure and provided key insights into the metabolic capabilities of inhabitant organisms, microcosm study confirmed that the supply of extraneous N as nitrate allows this community to make use of such biostimulatory factor thereby boosting the utilization of hydrocarbons.

## Author Contributions

JS performed the major experiments. PS and SK conceived and designed the work. JS, PS, and SK were responsible for manuscript preparation. AR and PS were responsible for sampling the oily waste sludge from refinery. AG, AD, and BM were responsible for optimization of PCR conditions and analysis of functional genes. JS, AG, and AD performed bioinformatic analysis using QIIME pipeline for microbial community analysis. PB and AM were responsible for GC-MS analysis and data interpretation.

## Conflict of Interest Statement

The authors declare that the research was conducted in the absence of any commercial or financial relationships that could be construed as a potential conflict of interest.

## Footnotes

1
http://www.expasy.org/tools/dna.html
