# Corrigendum: Biostimulation of indigenous microbial community for bioremediation of petroleum refinery sludge

**DOI:** 10.3389/fmicb.2025.1551928

**Published:** 2025-03-20

**Authors:** Jayeeta Sarkar, Sufia K. Kazy, Abhishek Gupta, Avishek Dutta, Balaram Mohapatra, Ajoy Roy, Paramita Bera, Adinpunya Mitra, Pinaki Sar

**Affiliations:** ^1^Department of Biotechnology, Indian Institute of Technology Kharagpur, Kharagpur, India; ^2^Department of Biotechnology, National Institute of Technology Durgapur, Durgapur, India; ^3^School of Bioscience, Indian Institute of Technology Kharagpur, Kharagpur, India; ^4^Department of Agricultural and Food Engineering, Indian Institute of Technology Kharagpur, Kharagpur, India

**Keywords:** petroleum refinery sludge, microbial community, bioremediation, biostimulation, next generation sequencing

In the published article, there was an error in the legend for **Supplementary Figure S4** as published. There was a typographical error in the legend causing S4C to be referred to as S4G. Chromatograms shown earlier were rescaled, which are now replaced with original chromatograms. The legend has been updated accordingly.

In the published article, there was an error in Figure 2, with the legend “Gas chromatogram for GR3, U and N samples.” In the original figure, the chromatogram of N_30 was inadvertently duplicated in place of N_90. The correct chromatogram has been restored and updated in the revised Figure 2. The corrected Figure 2 and its caption are provided here.

**Figure 2:**
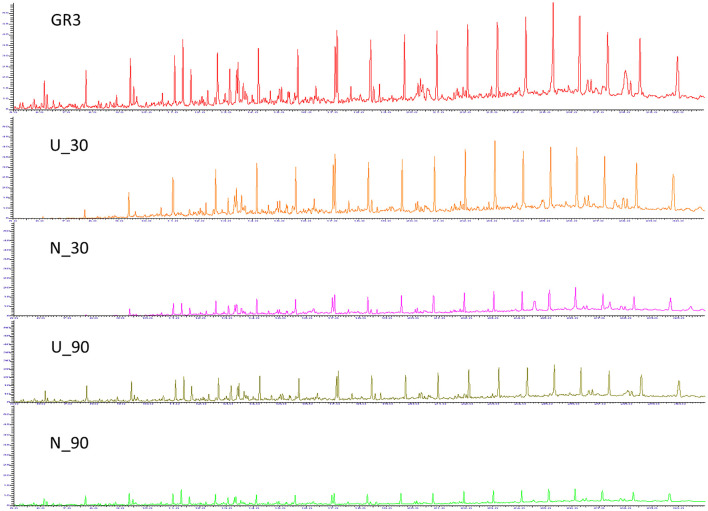
Gas chromatogram for GR3, U and N samples. GC-FID chromatograms showing constituent hydrocarbons after 30 and 90 days of microcosm incubation with or without nitrate amendment (U and N). Peaks have been assigned to carbon chain length corresponding to TPH standard (C10–28).”

In the published article, there was an error in Figure 3, with the legend “Denaturing gradient gel electrophoresis of microcosm samples.” Figure 3A showed the DGGE banding patterns of the studied samples and is comprised of lanes of the same gel which were spliced from the original gel image and arranged to match the UPGMA clustering done subsequently. Unfortunately, during the compilation of this gel image, lane P was duplicated as NS, and there was some mistake in the lane N as well. We are sorry for the oversight. The image has now been recompiled and all lanes of Figure 3A have been corrected. Re-analysis of the revised Figure 3A produced a change in the UPGMA clustering pattern and Figure 3B has been replaced accordingly. Based on the outcome of UPGMA re-analysis, the concerned results have been updated. The corrected Figure 3 and its caption are provided here.

**Figure 3:**
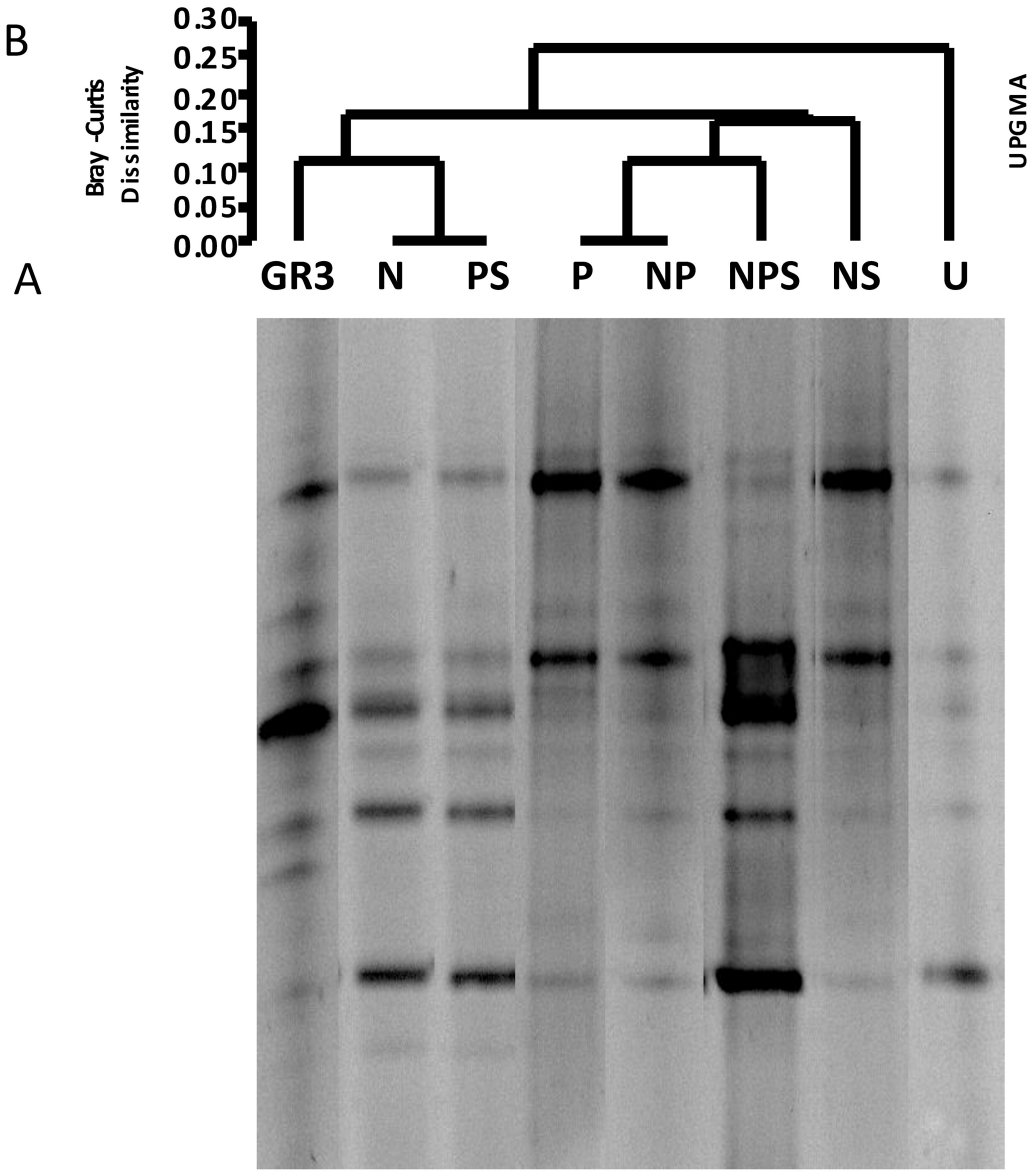
Denaturing gradient gel electrophoresis of microcosm samples. DGGE patterns of bacterial communities from microcosms with various amendments **(A)** and Bray–Curtis cluster analysis among the DGGE band patterns **(B)**. No amendment control (U) was also included. For Bray–Curtis analysis presence or absence of bands at corresponding positions was considered”.

In the published article, there was an error in Figure 3, as explained previously. In light of the rectification of this figure, the results inferred from it have also been updated.

A correction has been made to **Results**, *Shift in Microbial Community with Amendments* paragraph number one.

This sentence previously stated:

“However, the UPGMA on presence and absence of bands showed a relationship between the treatments (Figure 3B). Presence of two distinct clades at a similarity index of 0.25 to 0.2 showed a clear clustering of band patterns among GR3, N, P, PS (clade A) and U, NP, NS, NPS (clade B) samples. The result also indicated a distinct change in community composition following incubation within the microcosm without any treatment (U) which was consistent even for NP, NS and NPS amendments. Interestingly, even after incubating with N or P or PS, the community compositions were more similar to the original one (Figure 3A).”

The corrected sentence appears below:

“However, the UPGMA on presence and absence of bands showed a relationship between the treatments (Figure 3B). Presence of two distinct clades at a dissimilarity index of 0.15 to 0.20 showed a clustering of band patterns among GR3, N, PS (clade A) and P, NP, NPS, NS (clade B) samples with U cladding separately. The result also indicated a distinct change in community composition following incubation within the microcosm without any treatment (U). Interestingly, even after incubating with N or PS, the community compositions were more similar to the original one, relative to the other treatments (Figure 3A)”

The authors apologize for these errors and state that this does not change the scientific conclusions of the article in any way. The original article has been updated.

