# Supplementary material for: Biostimulation of Indigenous Microbial Community for Bioremediation of Petroleum Refinery Sludge
Source: Front Microbiol. 2016 Sep 21;7:1407. doi: 10.3389/fmicb.2016.01407 (PMC5030240; doi:10.3389/fmicb.2016.01407)
Supplement: Supplementary file 1 [file Data_Sheet_1.DOC]

Table S1. Summary of clones of functional genes

| **Functional gene** | **Clone ID** | **Protein family affiliated to** | **Closest classified relative (accession no.)** | **% identity** | **Phylogenetic affiliation** |
| --- | --- | --- | --- | --- | --- |
| *nif*H | HG_5 | Fe-nitrogenase protein | *Methanosarcinaacetovorans* (WP 011021640.1) | 92 | Archae*(Methanomicrobia)* |
|  | HN_2 | nitrogenase | *Methanocellapaludicola* (BAI60977.1) | 87 | Archae*(Methanomicrobia)* |
|  | HG_3 | nitrogenase | *Methanolineatarda* (WP 007315506.1) | 99 | Archae*(Methanomicrobia)* |
|  | HN_4 | Nitrogenase iron protein | *Methanosarcinaacetovorans* (WP 011021640.1) | 92 | Archae*(Methanomicrobia)* |
| *nar*G | nGN_3 | Thioredoxinreductase | *Azovibriorestrictus* (WP 026687569.1) | 62 | Bacteria *(Beta-Proteobacteria)* |
|  | NG1_7 | Methyltransferase protein | *Parcubacteria* (KKU86874.1) | 93 | *Bacteria* |
|  | NG1_3 | Thioredoxinreductase | *Hydrocarbinophagaeffusa* (WP_007187402.1) | 48 | Bacteria *(Gamma Proteobacteria)* |
| *alk*B | AG_1 | ATPase AAA | *Methanobacteriumformicicum* (WP_048073670.1) | 95 | Archae*(Methanomicrobia)* |
|  | AG_2 |  | *Methanobacteriumformicicum* (WP_048073670.1) | 94 | Archae*(Methanomicrobia)* |
|  | AN_1 |  | *Methanobacterium lacus* (WP_013645866.1) | 92 | Archae*(Methanomicrobia)* |
|  | AN_22 |  | *Methanobacteriumformicicum* (WP_048073670.1) | 95 | Archae*(Methanomicrobia)* |
| *bss*A | GL_3 | heat-shock protein HtpX | *Methanosaetaconcilii*(WP_013718019) | 74 | Archae *(Methanomicrobia)* |
|  | GL_10 | hypothetical protein XU14_C0027G0006 | *Armatimonadetes* bacterium CSP1-3 (sediment metagenome)(KRT7686) | 56 |  |
|  | GL_20 | heat-shock protein HtpX | *Methanosaetaconcilii*(WP_013718019) | 74 | Archae *(Methanomicrobia)* |
|  | GL_22 | methyltransferaseGidB | γ-Proteobacterium HTCC5015 (WP_008283933.1) | 58 | Bacteria *(Gamma Proteobacteria)* |
| *mcr*A | MN_2 | methyl coenzyme M reductase alpha subunit | *Methanolineatarda*NOBI-1 (BAF56441 ) | 96 | Archae *(Methanomicrobia)* |
|  | MN_5 |  | uncultured archaeon (AFA53815) | 99 | Archae |
|  | MG_3 |  | uncultured methanogenic archaeon (AAX84590) | 98 | Archae |
|  | MG_4 |  | uncultured archaeon (AFA53815) | 99 | Archae |
| *dsr*B | DG_2 | Sulphite reductase | *Peptococcaceae* bacterium BRH_c4b (KJS17143.1) | 97 | Bacteria *(Firmicutes)* |
|  | DG_6 | dissimilatorysulfitereductase subunit B | uncultured sulfate-reducing bacterium (CAJ84859.1) | 73 | Bacteria (metagenome of marine sediment) |
|  | DN_1 | dissimilatorysulfitereductase | Uncultured bacterium (ALA23410.1) | 94 | Bacteria |
|  | DN_2 | dissimilatorysulfitereductase beta subunit | uncultured sulfate-reducing bacterium (ALN40073) | 83 | Bacteria |

**(A)**

**(B)**

**(C)**

**(D)**

Figure S1. Utilization of 31 different C sources were monitored and substrates grouped into six different classes (according to Wang et al., 2012). Substrate utilization profile for (A) GR3 original oily sludge sample, (B) unamended control, (C) N amended control, (D) unpolluted soil. Error bars represent standard error of the mean (n=3).

Figure S2. Rarefaction curve showing the species richness in each of the four metagenomic amplicon library (GR3, unamended, N and NS amended microcosm samples)


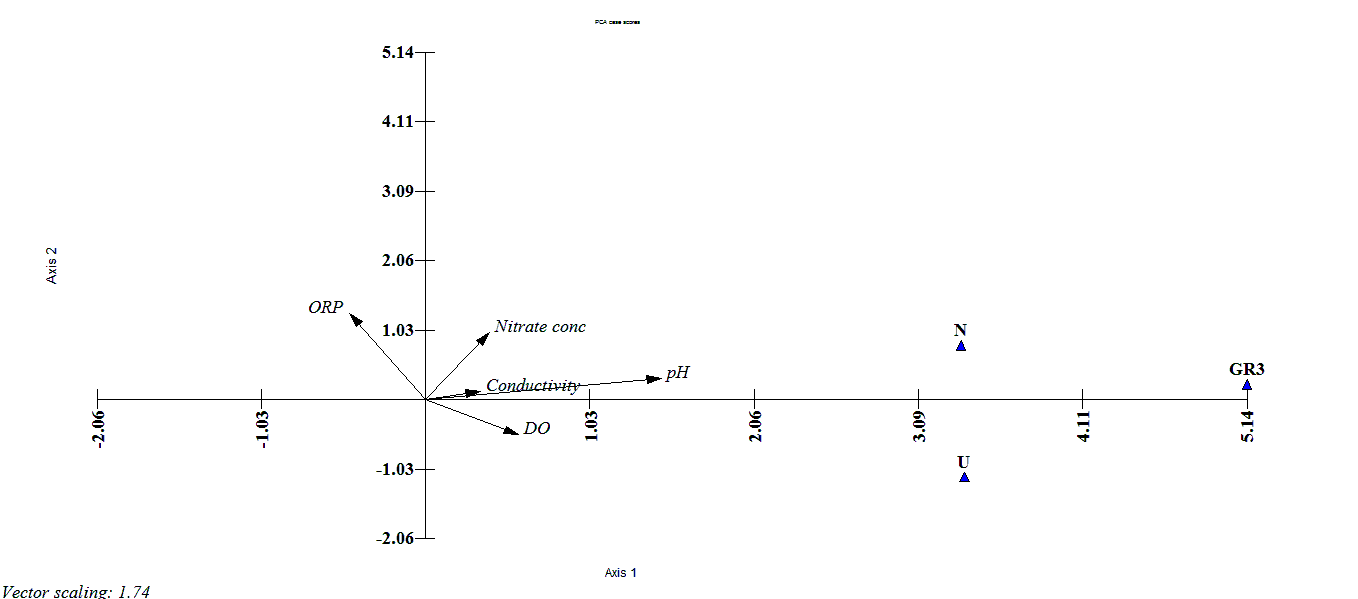


Figure S3. PCA plot showing relation between samples GR3, N and U based on physico-chemical parameters (DO, ORP, pH, conductivity, nitrate concentration).


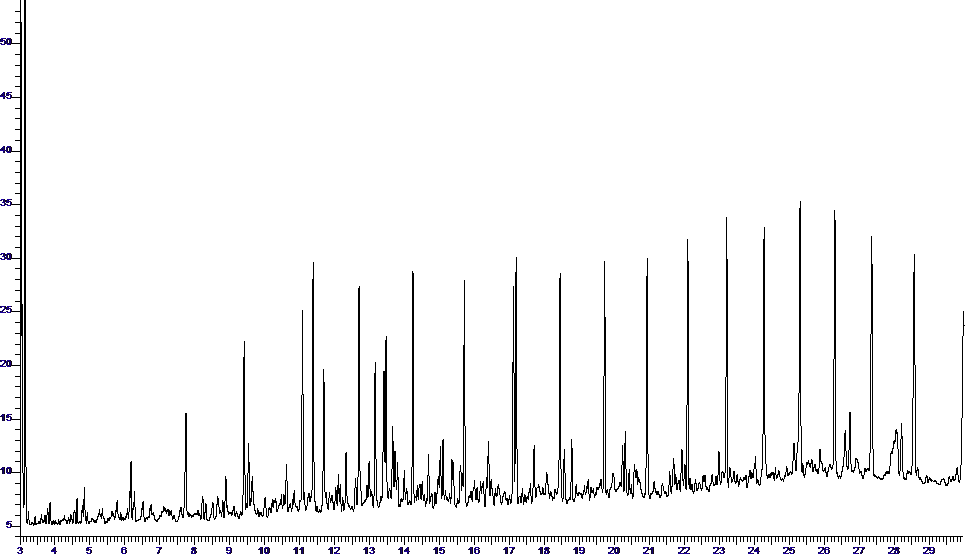

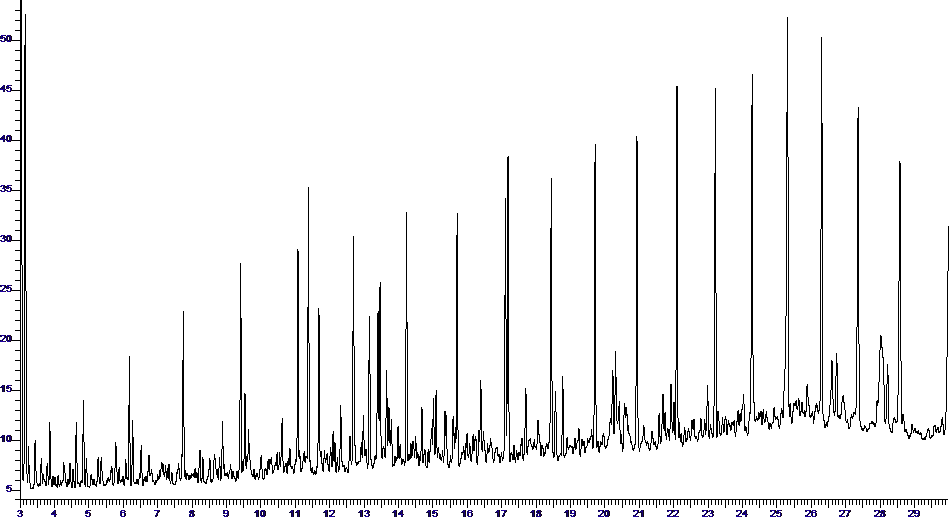

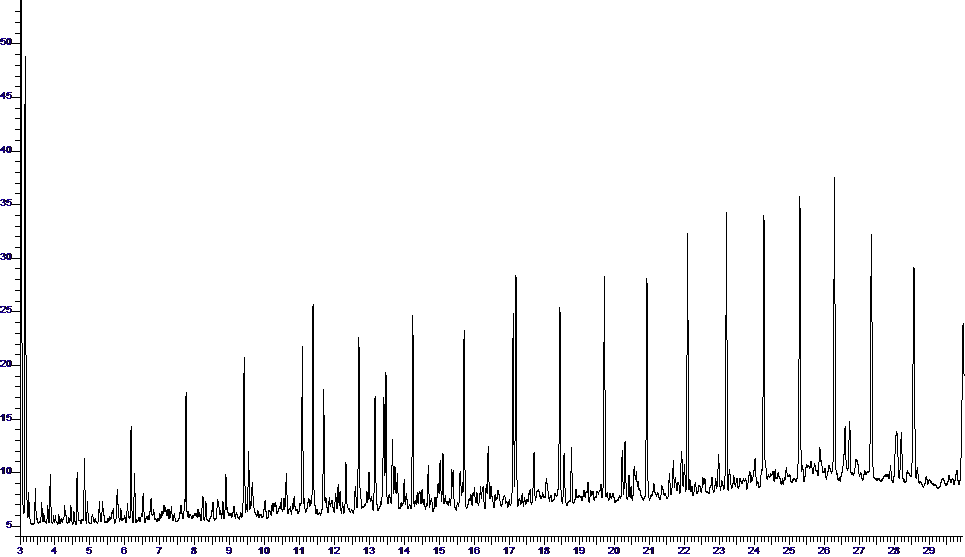

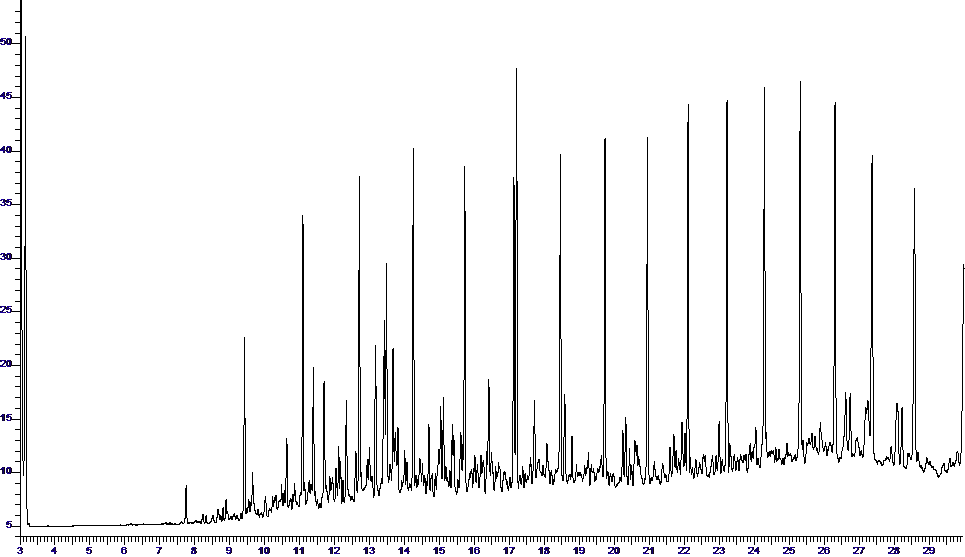

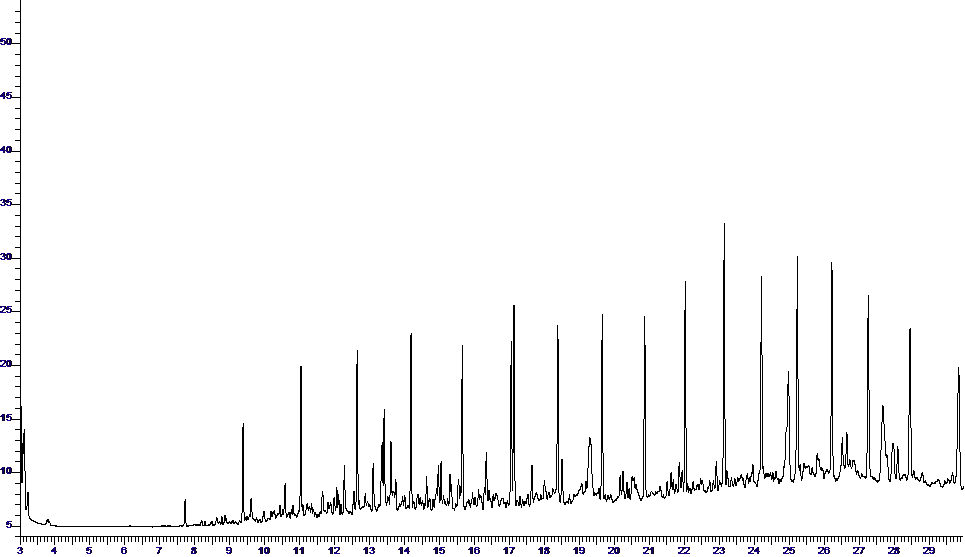

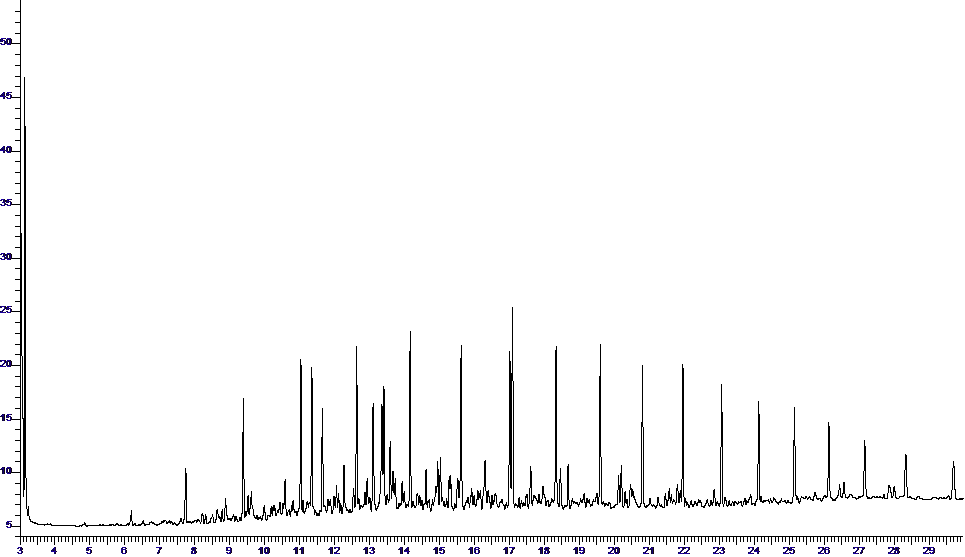


(A)

(F)

(E)

(D)

(C)

(B)

<C10

C11-16

C17-25

>C25

Figure S4. Gas chromatogram of selected samples, (A) original sludge before bioremediation treatment, (B, C) U after 21 and 90 days of incubation, (D)-(F) N after 21,60 and 90 days of incubation. Chromatograms show response variable (mV) on Y axis with a scale of 4-50 mV and retention time (3-30 mins) along X axis. Original chromatograms have been rescaled for better viewing purpose. Carbon chain length range was determined by comparing with known TPH mix 3 (Sigma Aldrich) standard retention times, run in same method as that of the sample and displayed at the top.
